# Bioactive Hydrogel–MOF Composites as Resistance-Modulating Wound Interfaces: Molecular Mechanisms and Rational Design for Chronic Wound Management

**DOI:** 10.3390/gels12080744

**Published:** 2026-08-20

**Authors:** Nallely G. Hernández-Hernández, Irving A. González-Lara, Lesly Katleya Usme-Duque, Lía A. Martínez-Berlanga, Grecia D. Ortíz-Hernández, María I. León-Campos, Bertha Puente-Urbina, Miguel A. Medina-Morales, Elan I. Loredo-Alcalá, Leopoldo J. Ríos-González, Thelma K. Morales-Martínez, Roberto Arredondo-Valdés, Adolfo Romero-Galarza, Lucía F. Cano-Salazar, Rebeca Betancourt-Galindo, María O. González-Díaz, Nayeli Rodríguez-Fuentes, Javier Enríquez-Medrano, Florentino Soriano-Corral, Raul Rosales-Ibáñez, Amairany Rodríguez-Navarrete, Denis A. Cabrera-Munguía, Jesús A. Claudio-Rizo

**Affiliations:** 1 Facultad de Ciencias Químicas, Universidad Autónoma de Coahuila, Ing. J. Cárdenas Valdez S/N, República, Saltillo 25280, Coahuila, Mexicokatleya_usme@uadec.edu.mx (L.K.U.-D.); lia.berlanga@uadec.edu.mx (L.A.M.-B.); lucia.cano@uadec.edu.mx (L.F.C.-S.); 2 Departamento de Horticultura, Universidad Autónoma Agraria Antonio Narro, Calz. Antonio Narro 1923, Buenavista, Saltillo 25315, Coahuila, Mexico; 3 Centro de Investigación en Química Aplicada (CIQA), Blvd. Enrique Reyna Hermosillo 140, San José de los Cerritos, Saltillo 25294, Coahuila, Mexico; 4 Center for Biodiversity Conservation and Ecology of Coahuila, Universidad Autónoma de Coahuila, Miguel Hidalgo 212, Zona Centro, Cuatro Ciénegas 27640, Coahuila, Mexico; 5 Centro de Investigación Científica de Yucatán (CICY), Calle 43 No. 130 × 32 y 34, Chuburná de Hidalgo, Mérida 97205, Yucatán, Mexiconayeli.rodriguez@cicy.mx (N.R.-F.); 6 Ministry of Science, Humanities, Technology and Innovation (SECIHTI), Av. Insurgentes Sur 1582, Crédito Constructor, Benito Juárez, Mexico City 03940, Mexico; 7 Tissue Engineering Laboratory and Translational Dentistry Clinic, Facultad de Estudios Superiores Iztacala, National Autonomous University of Mexico (UNAM), Tenayuca-Chalmita S/N, Cuautepec Barrio Bajo, Gustavo A. Madero, Mexico City 07239, Mexico; rosales_ibanez@unam.mx (R.R.-I.);

**Keywords:** chronic wounds, biopolymers hydrogels, metal–organic frameworks (MOFs), resistance-modulating wound interfaces, antimicrobial resistance, biofilm destabilization, efflux pump inhibitors (EPIs), horizontal gene transfer (HGT), quorum sensing (QS), microenvironment regulation

## Abstract

Chronic wounds are complex environments marked by persistent inflammation, oxidative stress, hypoxia, and conditions that favor antimicrobial resistance (AMR). Conventional antibiotics often fail due to bacterial persistence and the physicochemical barriers of the wound milieu. Biofilm-associated extracellular polymeric substances (EPS), efflux pump activity, quorum sensing (QS), and horizontal gene transfer (HGT) collectively drive antimicrobial tolerance and resistance dissemination, turning chronic wounds into reservoirs of multidrug-resistant pathogens. Consequently, emerging wound therapies demand multifunctional strategies that modulate the wound microenvironment while interfering with resistance-associated phenotypes. Hydrogel–metal–organic framework (MOF) composites have been explored as multifunctional interfaces that combine extracellular matrix-mimetic properties, tunable porosity, stimuli-responsiveness, and controlled therapeutic delivery with the bioactive functions of MOFs. Depending on their composition and architecture, these systems may exert antimicrobial and antibiofilm effects through ionic, electrostatic, osmotic, catalytic, and oxidative mechanisms, while also influencing ROS levels, inflammation, angiogenesis, and local drug transport. However, antimicrobial activity alone does not equate to resistance modulation. Evidence for direct effects on efflux systems, resistance phenotypes, or HGT remains inconsistent across reported platforms. This review critically examines representative hydrogel–MOF systems for chronic wound applications, comparing their composition, physicochemical properties, biological functions, proposed resistance-related mechanisms, advantages, limitations, and current level of evidence. We emphasize distinguishing experimentally demonstrated resistance-modulating effects from mechanistically proposed functions, and identifying design trade-offs and evidence gaps that must be addressed to develop wound interfaces capable of both supporting tissue regeneration and improving infection control.

## 1. Introduction

### 1.1. Chronic Wounds and AMR

Chronic wounds pose a major healthcare challenge due to persistent inflammation, impaired tissue regeneration, and high susceptibility to infection [1]. These recalcitrant lesions are characterized by biofilm persistence, oxidative imbalance, hypoxia, altered pH, and dysregulated immune responses [2]. Such conditions promote the emergence and dissemination of AMR, turning chronic wounds into dynamic reservoirs of multidrug-resistant microorganisms [3]. Within these environments, bacterial communities embedded in EPS matrices show enhanced tolerance to antibiotics and host immune defenses, while high cellular density and prolonged microbial interactions facilitate HGT, QS, and the evolution of adaptive resistance phenotypes [4]. Consequently, conventional antibiotic-centered therapies often fail to eradicate chronic infections, particularly when the biofilm and wound microenvironment remain unaltered [5]. Current therapeutic approaches have traditionally focused on bacterial elimination through systemic antibiotics, antiseptics, or metallic agents [6]. However, increasing evidence indicates that bacterial persistence in chronic wounds is sustained not only by microbial abundance, but also by the ecological and physicochemical conditions that stabilize biofilms, promote efflux-mediated tolerance, regulate oxidative stress, and facilitate resistance dissemination [7]. Rather than targeting bacterial burden alone, effective intervention requires simultaneous consideration of the wound microenvironment, biofilm architecture, and resistance-associated phenotypes.

### 1.2. Biopolymer Hydrogels as Wound Interfaces

This paradigm shift has driven growing interest in biomaterial-based therapeutic strategies that actively modulate the wound microenvironment rather than exerting bactericidal activity alone [8]. In this context, biopolymer hydrogels have emerged as promising wound interfaces due to their extracellular matrix (ECM)-like architecture, high hydration capacity, tunable porosity, and adaptable physicochemical properties. Beyond serving as passive dressings, hydrogels can dynamically regulate local pH, ROS, oxygen diffusion, inflammatory signaling, and cellular responses associated with tissue repair [9]. Moreover, their ability to incorporate bioactive compounds, antimicrobial agents, and stimuli-responsive functionalities enables multifunctional systems capable of promoting both infection control and tissue regeneration [10]. However, most natural biopolymers lack intrinsic bactericidal activity and, due to their high-water uptake and chemical composition, may promote microbial growth [11].

### 1.3. MOFs: Structure, Properties and Relevance for Wound Healing

MOFs are a rapidly expanding class of crystalline porous coordination materials formed by the self-assembly of metal ions or clusters with multidentate organic ligands [12]. Their exceptionally high surface area, tunable pore architecture, controllable chemistry, and ability to encapsulate or release therapeutic cargoes have attracted increasing attention for biomedical applications, including drug delivery, biosensing, tissue engineering, and wound management [13]. These characteristics distinguish MOFs from conventional inorganic nanoparticles by providing both structural versatility and programmable biological functionality. MOFs can be constructed from a wide variety of metal ions, whose biological functions differ considerably. Essential metal ions such as Zn, Cu, Fe, Co, and Mg participate in physiological processes relevant to tissue repair [13]. For example, Zn serves as a cofactor for matrix metalloproteinases involved in ECM remodeling; Cu promotes angiogenesis and vascular maturation; Fe participates in oxygen transport and redox metabolism; Co can activate hypoxia-associated signaling pathways; and Mg supports cellular proliferation and neovascularization [14]. In contrast, Ag has no known physiological role in human tissues but is widely incorporated into MOFs because of its potent broad-spectrum antimicrobial activity, primarily through disruption of bacterial cell envelopes and membrane integrity [15]. Consequently, depending on their metal composition, MOFs can exhibit distinct antibacterial, pro-angiogenic, immunomodulatory, and redox-regulating properties that may influence wound healing [16]. Importantly, MOFs can function not only as drug carriers but also as molecular signal interceptors that modulate bacterial communication, oxidative stress, biofilm stability, and adaptive resistance mechanisms [17]. However, these functions depend heavily on the metal node, organic linker, framework stability, degradation kinetics, and local wound conditions. Therefore, MOFs should not be considered a uniform class of bioactive materials, and their potential advantages must be evaluated against possible limitations such as uncontrolled metal-ion release, cytotoxicity, limited aqueous stability, and insufficient evidence for direct resistance modulation.

### 1.4. Rationale for Integrating MOFs into Hydrogels

The integration of MOFs within hydrogel networks can provide complementary functions, including localized therapeutic delivery, improved material stability, and environmentally responsive release [18]. However, these advantages depend strongly on the properties of both components and are not necessarily achieved in every hydrogel–MOF formulation. Hydrogels primarily provide hydration, tissue contact, local retention, and control over mass transport, whereas MOFs contribute to tunable porosity, cargo loading, catalytic activity, and metal-ion-mediated biological effects. Their integration may therefore improve therapeutic performance when these functions are complementary; conversely, the hydrogel network may alter MOF degradation, diffusion, and ion-release kinetics, potentially limiting bioavailability or introducing additional safety and manufacturing constraints.

Emerging studies have proposed that hydrogel–MOF composites may interfere with resistance-associated processes, including efflux activity, EPS stabilization, QS, and HGT within biofilm communities [19]. Nevertheless, the strength of evidence varies substantially among these mechanisms. Effects such as reduced bacterial viability, ROS generation, membrane disruption, or EPS destabilization should not, by themselves, be interpreted as direct evidence of resistance modulation. Therefore, potential changes in antibiotic susceptibility or resistance-associated phenotypes should be evaluated separately from general antibacterial activity. This distinction is particularly important when interpreting the proposed effects of controlled ion release, ROS generation, osmotic or electrostatic interactions, and stimuli-responsive therapeutic delivery [20].

To date, much of the literature on hydrogel–MOF composites has emphasized their synthesis, physicochemical characterization, therapeutic loading, and antibacterial performance, with metal-ion release and ROS generation frequently identified as important contributors to microbial inactivation [21,22]. However, antibacterial activity alone does not establish modulation of antimicrobial resistance. The emerging interest in hydrogel–MOF composites as resistance-modulating wound interfaces therefore requires a more critical assessment of whether these systems can directly or indirectly influence biofilm-associated tolerance, efflux activity, antibiotic susceptibility, resistance-associated phenotypes, or resistance dissemination [23]. Importantly, the extent to which these effects are experimentally demonstrated varies among the reported platforms, and several proposed mechanisms remain based on indirect or mechanistic evidence.

Compared with conventional responsive wound dressings, the integration of MOFs into hydrogel networks can introduce additional physicochemical and biological functions. However, these properties do not necessarily translate into superior therapeutic performance. Their effects depend on the MOF composition, coordination chemistry, framework stability, hydrogel architecture, release kinetics, and local wound conditions. Accordingly, the critical question is not whether hydrogel–MOF composites exhibit multiple functions, but whether the integration of both components provides measurable advantages over the corresponding hydrogel or MOF alone while maintaining adequate biocompatibility, stability, and therapeutic control [14].

Accordingly, this review critically examines and compares representative bioactive hydrogel–MOF systems for chronic wound management, with emphasis on their composition, physicochemical characteristics, biological functions, mechanisms of biofilm destabilization, evidence for resistance modulation, microenvironmental regulation, and translational limitations. Particular attention is given to distinguishing experimentally demonstrated effects from mechanistically proposed functions and to evaluating the advantages, limitations, and design trade-offs associated with different hydrogel–MOF architectures. This comparative framework is used to identify current evidence gaps and define design considerations for next-generation wound interfaces that aim to simultaneously support tissue regeneration and improve infection control. The conceptual framework discussed in this review, including the multiscale mechanisms involved in biofilm destabilization, resistance modulation, and microenvironmental regulation by hydrogel–MOF systems, is summarized in Figure S1.

## 2. Chronic Wounds as Reservoirs of Antimicrobial Resistance Development

Chronic wounds are characterized by persistent inflammation, impaired tissue repair, and recurrent microbial colonization, creating pathological microenvironments that promote infection, biofilm persistence, and AMR. Unlike acute wounds, these lesions fail to progress through the normal healing cascade, resulting in prolonged inflammation, delayed regeneration, and increased healthcare burden, including pain, disability, limb amputation, prolonged hospitalization, and elevated medical costs [7]. These multifactorial conditions underscore the need for multifunctional therapeutic platforms capable of simultaneously controlling infection, modulating the wound microenvironment, and promoting tissue regeneration.

### 2.1. Microbial Environment and Biofilm Persistence

Chronic wounds harbor diverse polymicrobial communities dominated by *Staphylococcus*, *Pseudomonas*, *Enterobacter*, and anaerobic bacteria, which frequently establish synergistic interactions that enhance biofilm stability, bacterial persistence, and virulence [24]. Biofilms are detected in approximately 60–70% of chronic wounds and consist of microorganisms embedded within an EPS matrix that restricts antimicrobial penetration, protects bacteria from host immune defenses, and sustains chronic inflammation and metabolic heterogeneity [25]. As illustrated in Figure S2, these biofilm-associated microenvironments facilitate bacterial persistence and the dissemination of resistance-associated phenotypes.

Because biofilm behavior is strongly influenced by polymicrobial interactions, oxygen gradients, pH fluctuations, host-derived biomolecules, and dynamic wound conditions, the performance of antimicrobial biomaterials—including hydrogel–MOF composites—cannot be directly extrapolated from simplified in vitro models [26]. Although experimental wound models provide valuable mechanistic insights, they only partially reproduce the complexity of clinical chronic wounds. Consequently, the antibiofilm efficacy, degradation behavior, and translational potential of hydrogel–MOF systems should be interpreted cautiously and validated under clinically relevant conditions.

### 2.2. Clinical Implications and Therapeutic Challenges

Biofilm-associated tolerance and AMR markedly reduce the effectiveness of conventional wound management strategies, including debridement, antiseptics, and systemic antibiotics, which often decrease bacterial burden without eradicating mature biofilms [27]. As a result, recurrent infections, persistent inflammation, delayed healing, and an increased risk of tissue necrosis or amputation remain major clinical challenges.

Chronic wound infections also impose a substantial socioeconomic burden by increasing hospitalization rates, healthcare costs, and long-term patient morbidity, while serving as reservoirs for AMR dissemination within healthcare settings [24]. Consequently, effective wound management requires therapeutic strategies capable of targeting not only bacterial viability but also the physicochemical and ecological mechanisms underlying biofilm persistence and resistance evolution. An overview of the major AMR mechanisms, associated microorganisms, and their clinical implications is presented in Table 1. These challenges have stimulated the development of multifunctional biomaterials, particularly hydrogel–MOF composites, which combine localized therapeutic delivery, biofilm modulation, and tissue-regenerative functions within a single platform. However, current evidence is still largely derived from controlled experimental models, and important challenges remain regarding efficacy in polymicrobial infections, long-term biosafety, scalability, and clinical translation [28].

### 2.3. Emerging Strategies to Counteract AMR in Chronic Wounds

To reduce antibiotic dependence and overcome biofilm-associated resistance, increasing attention has been directed toward multifunctional biomaterials capable of simultaneously controlling infection, modulating the wound microenvironment, and promoting tissue regeneration. Functional hydrogels incorporating cationic polymers, metallic nanoparticles, or metal oxide nanostructures have demonstrated antibiofilm, anti-inflammatory, and regenerative properties while enabling localized delivery of therapeutic agents [36,37].

Among these approaches, hydrogel–MOF composites have emerged as particularly promising platforms. This integration enables localized retention, controlled release of bioactive species, and modulation of mass transport and therapeutic kinetics, generating synergistic functions that cannot be achieved by either component alone. However, composite performance depends on the interplay between MOF composition, framework stability, hydrogel architecture, and the wound microenvironment. Consequently, antibacterial activity alone should not be interpreted as evidence of AMR modulation; instead, hydrogel–MOF systems should be evaluated according to their ability to improve biofilm disruption, antibiotic susceptibility, tissue regeneration, and other resistance-associated outcomes relative to their individual components [28].

Other emerging strategies, including bacteriophage therapy, matrix-degrading enzymes, antimicrobial peptides, electroceutical dressings, and QS inhibitors, are also being explored to destabilize biofilms and limit AMR dissemination [38]. Collectively, these approaches reflect a shift from pathogen eradication toward modulation of the wound microenvironment and resistance-associated phenotypes. Nevertheless, direct comparisons among therapeutic platforms remain limited by differences in experimental models, bacterial species, material composition, and evaluation criteria, highlighting the need for standardized studies to define the specific advantages of hydrogel–MOF composites over existing wound management strategies [28].

## 3. Bioactive Hydrogels as Microenvironment-Modulating Interfaces

### 3.1. Structural and Functional Basis of Bioactive Hydrogels

Bioactive hydrogels are three-dimensional hydrated polymeric networks that mimic key structural and physicochemical features of the ECM, providing a favorable microenvironment for nutrient diffusion, gas exchange, cellular infiltration, and localized therapeutic delivery [39].

The physicochemical properties of hydrogels can be tailored through physical or chemical crosslinking, enabling control over swelling behavior, degradation, mechanical stability, permeability, and cell–material interactions [39]. This tunability also facilitates the incorporation of therapeutic agents and functional nanomaterials, allowing hydrogels to operate as multifunctional wound interfaces. When integrated with MOFs, however, hydrogel networks play a role beyond simple encapsulation. Polymer chemistry, crosslinking density, and network architecture govern MOF distribution, degradation, mass transport, and the release of bioactive species, ultimately determining the performance of the composite. Consequently, the therapeutic behavior of hydrogel–MOF systems results from the synergistic interaction between both components rather than from the individual properties of either the hydrogel or the MOF alone.

### 3.2. Microenvironmental Regulation in Chronic Wounds

Chronic wounds, particularly diabetic ulcers, exhibit dysregulated microenvironments characterized by hyperglycemia, hypoxia, acidic pH, excessive ROS, persistent inflammation, and impaired angiogenesis, all of which contribute to delayed healing, biofilm persistence, and tissue damage [40]. Consequently, advanced wound therapies increasingly aim to restore microenvironmental homeostasis in addition to controlling microbial burden.

Stimuli-responsive hydrogels capable of responding to pH, glucose, ROS, enzymes, or temperature provide adaptive platforms for localized therapeutic delivery by regulating degradation behavior and the controlled release of antioxidants, anti-inflammatory agents, angiogenic factors, and antimicrobials [40,41]. When integrated with MOFs, these adaptive properties can generate synergistic effects that simultaneously modulate the wound microenvironment and support tissue regeneration. Nevertheless, the magnitude and durability of these therapeutic responses remain dependent on hydrogel composition, MOF characteristics, stimulus responsiveness, and the pathological conditions of the wound.

### 3.3. Immunomodulation and Regenerative Signaling

The physicochemical properties of hydrogels, including stiffness, surface chemistry, and degradation behavior, regulate mechanotransduction, immune-cell responses, and tissue regeneration [42]. In particular, hydrogel-mediated modulation of macrophage polarization from the pro-inflammatory M1 phenotype toward the pro-regenerative M2 phenotype has emerged as an important strategy to attenuate chronic inflammation and promote tissue remodeling [43].

The incorporation of bioactive molecules, such as VEGF, IL-10, growth factors, and polyphenolic compounds, further enhances angiogenesis, collagen deposition, and re-epithelialization, creating regenerative microenvironments that support wound healing [28]. In hydrogel–MOF composites, these regenerative processes may act synergistically with MOF-mediated ion release, catalytic activity, and antimicrobial functions. However, the therapeutic outcome depends on the specific hydrogel formulation, MOF composition, and experimental model, emphasizing the need to evaluate the contribution of each component to the overall biological performance of the composite.

### 3.4. Beyond Antibacterial Action: Microenvironment-Driven Therapeutic Strategies

Conventional treatment of infected wounds has primarily relied on antibiotics, antiseptics, and metallic ions to reduce bacterial burden. However, these approaches often show limited efficacy against mature biofilms and may contribute to AMR through sustained selective pressure [27]. Consequently, advanced biomaterial-based therapies increasingly focus on restoring the dysregulated wound microenvironment in addition to controlling microbial growth.

Stimuli-responsive hydrogels can modulate key pathological features of chronic wounds, including oxidative stress, pH imbalance, inflammatory signaling, QS, and biofilm stability, thereby promoting tissue repair while improving local infection control [40,44]. As summarized in Table 2, these strategies can be broadly categorized into pathogen-centered and microenvironment-modulating approaches. When integrated with MOFs, hydrogels can enable multifunctional platforms that simultaneously regulate infection, biofilm persistence, and tissue regeneration. Nevertheless, reductions in bacterial burden alone should not be interpreted as evidence of resistance modulation, which requires direct evaluation using mechanism-specific endpoints.

Despite these advances, important challenges remain for hydrogel–MOF composites, including optimization of stimuli responsiveness, material stability, therapeutic release, biodegradation, and long-term biocompatibility. Achieving an appropriate balance among these parameters will be essential for translating multifunctional hydrogel–MOF systems into clinically relevant wound therapies [28].

### 3.5. Transition Toward Resistance-Modulating Interfaces

The concept of resistance-modulating wound interfaces extends the role of bioactive hydrogels beyond passive or antimicrobial dressings toward multifunctional platforms capable of simultaneously regulating infection, biofilm persistence, inflammatory signaling, and tissue regeneration [46,51]. Through localized therapeutic delivery and stimuli-responsive release, these systems may reduce reliance on systemic antibiotics while creating conditions less favorable for resistance-associated phenotypes [52]. However, improvements in antimicrobial activity or wound healing should not be interpreted as direct evidence of antimicrobial resistance modulation, which requires mechanism-specific validation.

**Figure 1:**
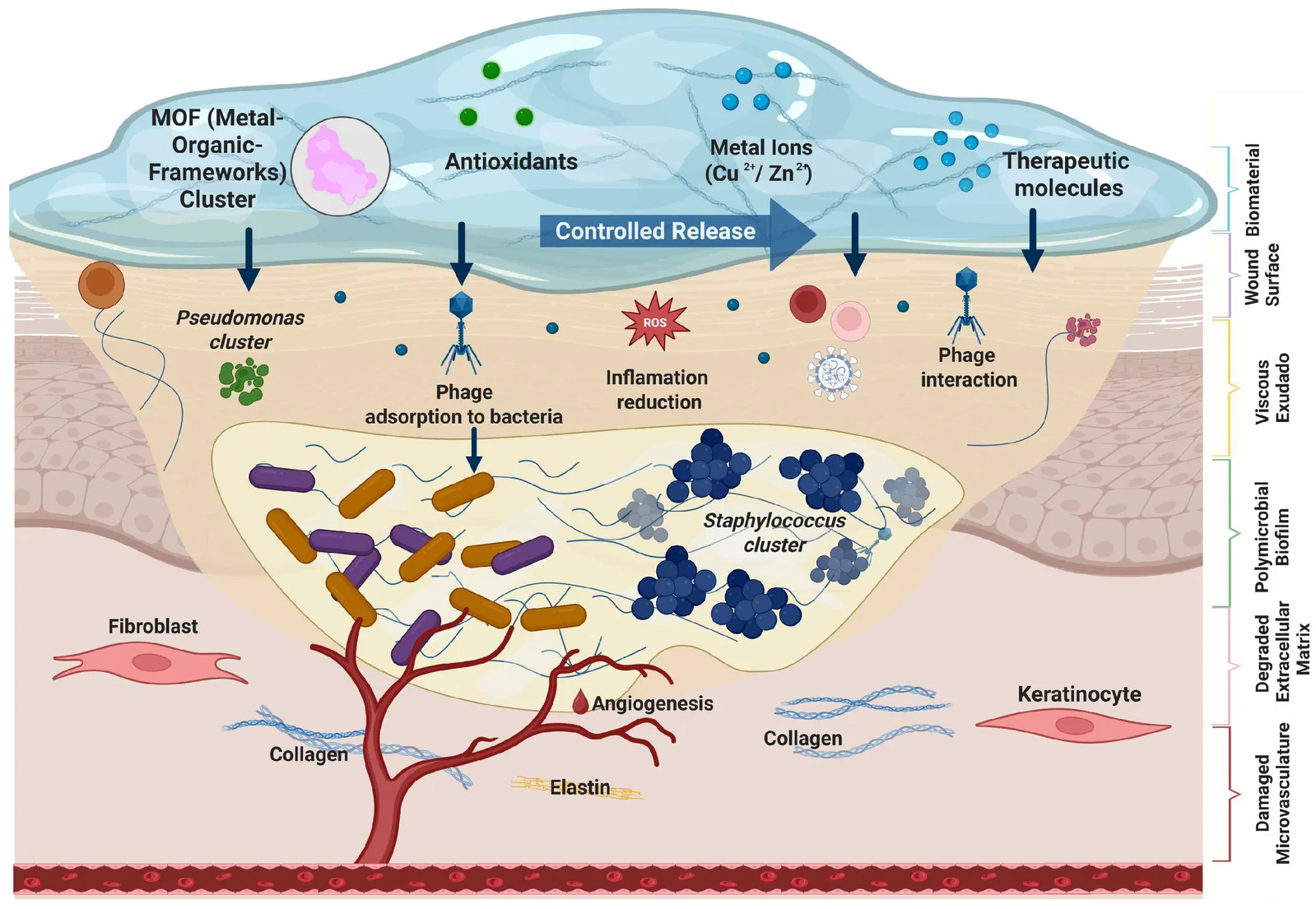
Schematic representation of microenvironment modulation in chronic wounds by biopolymer hydrogel–MOF systems. Created in BioRender. Rizo, J. (2026) https://BioRender.com/aikyh19 (accessed on 31 July 2026).

The integration of hydrogels with MOFs further expands these capabilities by combining adaptive hydrogel networks. This synergistic design enables simultaneous modulation of the wound microenvironment and localized therapeutic delivery, providing a rational strategy for multifunctional wound management. The multifactorial mechanisms involved in microenvironmental regulation by hydrogel–MOF systems are summarized in Figure 1.

Despite these advances, important challenges remain regarding long-term material stability, degradation behavior, reproducibility, host compatibility, and performance under the dynamic conditions of chronic wounds [53]. Addressing these limitations will be essential to determine whether hydrogel–MOF composites can progress from multifunctional experimental platforms toward clinically relevant resistance-modulating wound interfaces.

## 4. MOFs as Molecular Signal Interceptors

**Figure 2:**
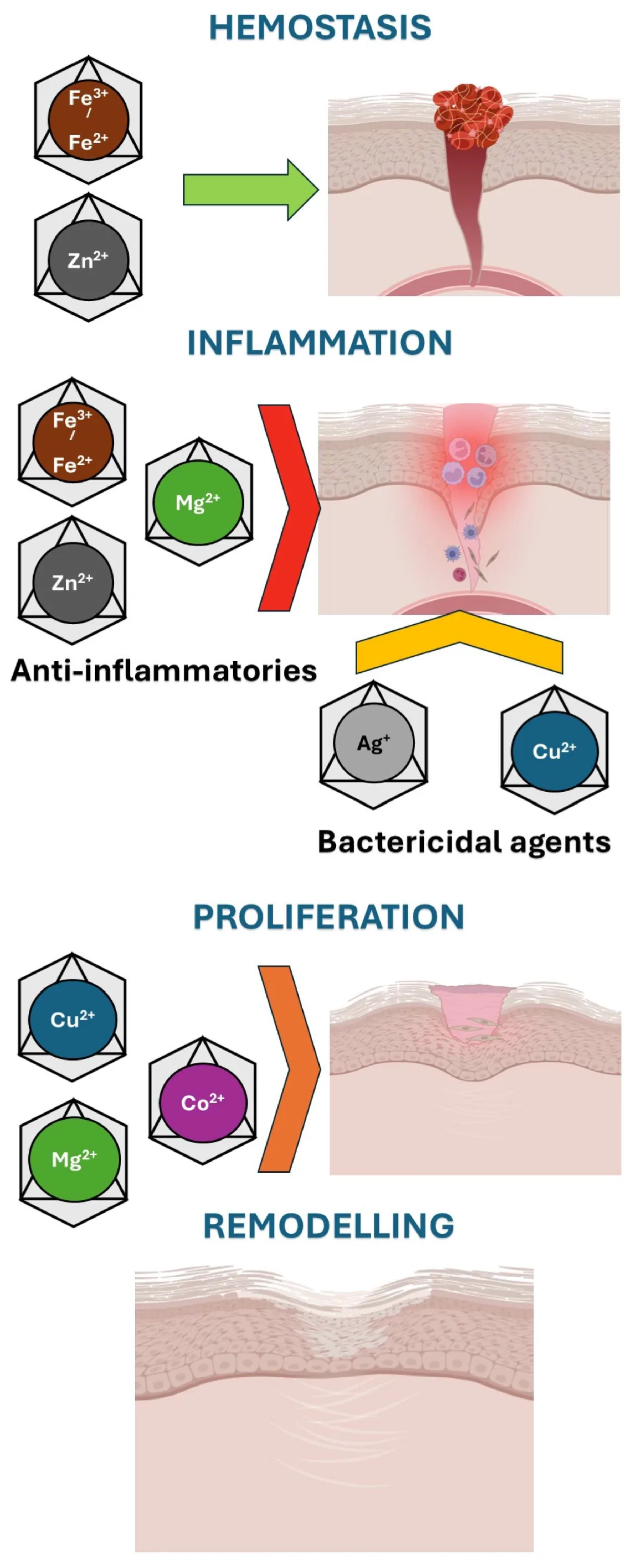
MOFs’ participation in wound healing steps. Created in BioRender. Cabrera, D. (2026) https://BioRender.com/lql6bku (accessed on 31 July 2026).

MOFs have evolved from conventional drug carriers into multifunctional materials capable of actively modulating the biochemical and physicochemical processes that govern chronic wound progression (Figure 2) [53,54]. Consequently, the biological activity of MOFs arises from the combined contributions of both therapeutic cargo and framework-derived metal ions rather than from drug delivery alone.

When integrated into biopolymer hydrogels, these functions can be further enhanced through improved properties, generating synergistic therapeutic effects that cannot be achieved by either component independently [53,54]. Nevertheless, the biological performance of hydrogel–MOF composites remains highly dependent on MOF composition, metal coordination environment, ion-release kinetics, and the pathological conditions of the wound. Therefore, increased metal-ion availability should not be assumed to directly translate into improved therapeutic efficacy.

From this perspective, selected MOFs can be considered molecular signal interceptors because they have the potential to influence signaling pathways associated with inflammation, oxidative stress, angiogenesis, and microbial adaptation. However, distinguishing direct molecular pathway modulation from secondary microenvironmental effects or nonspecific cytotoxicity remains a major challenge for the field. Accordingly, Table S1 summarizes representative metal-based MOFs according to their structural characteristics, physicochemical properties, reported biological functions, therapeutic potential, and current mechanistic limitations relevant to molecular signal interception.

### 4.1. Metal-Dependent Mechanisms of Signal Modulation

The biological behavior of MOFs is primarily determined by the identity and coordination chemistry of their metal centers, which govern degradation behavior, ion release, catalytic activity, and interactions with cells, microorganisms, and the extracellular environment. Representative structural characteristics of the principal MOFs discussed in this review, including their metal nodes, organic linkers, coordination environments, secondary building units (SBUs), and pore architectures, are summarized in Table S2 and illustrated in Figure S3. These structural differences result in distinct biological functions, ranging from antimicrobial activity and redox regulation to angiogenesis and tissue regeneration, highlighting that metal ions should not be considered interchangeable therapeutic agents.

Silver-based MOFs: Representative Ag-based MOFs are constructed from Ag(I) coordination nodes linked by nitrogen- or carboxylate-containing ligands (Table S2; Figure S3). Their therapeutic activity is primarily attributed to the sustained release of Ag^+^, which interacts with thiol-containing proteins, disrupts respiratory metabolism and nucleic acid function, and promotes oxidative stress, thereby providing broad-spectrum antimicrobial activity against wound pathogens [15]. Compared with soluble silver salts, Ag-based MOFs enable more controlled ion release and have been combined with photothermal strategies to improve antibiofilm performance [15]. However, their biological efficacy depends on maintaining Ag^+^ concentrations within a therapeutic window, as excessive ion release may compromise host-cell viability. Consequently, rational framework design should prioritize controlled spatial and temporal silver delivery rather than maximizing ion release.

Copper-based MOFs: Representative Cu-based MOFs, particularly HKUST-1, are constructed from Cu_2_ paddlewheel secondary building units coordinated by benzene-1,3,5-tricarboxylate (BTC) ligands, generating highly porous frameworks with controlled cargo loading and Cu^2+^ release (Table S2; Figure S3) [54,55]. Unlike Ag-based systems, Cu-MOFs exhibit a dual therapeutic role by simultaneously promoting antimicrobial activity and tissue regeneration. Released Cu^2+^ participates in angiogenesis, ECM remodeling, macrophage polarization, and inflammatory regulation, while its redox activity generates ROS that contribute to bacterial elimination [54,55,56]. In addition, HKUST-1 and related Cu-MOFs have been explored as carriers for nitric oxide (NO), further enhancing angiogenic and regenerative signaling [16,55]. These multifunctional properties make Cu-based MOFs particularly attractive for hydrogel integration, where localized retention and controlled release can maximize therapeutic efficacy while minimizing oxidative stress and cytotoxicity associated with excessive Cu^2+^ or NO exposure.

Cobalt-based MOFs: Co-based MOFs, represented by ZIF-67, consist of Co^2+^ ions tetrahedrally coordinated by 2-methylimidazolate ligands to form a zeolitic framework structurally analogous to ZIF-8 (Table S2; Figure S3) [16]. Their principal therapeutic advantage lies in promoting angiogenesis through stabilization of hypoxia-inducible factor-1α (HIF-1α), which enhances the expression of VEGF and other pro-angiogenic mediators under hypoxic wound conditions [53,54]. Consequently, Co-MOFs have been investigated as multifunctional platforms that combine therapeutic cargo delivery with stimulation of vascular regeneration [16]. However, the biological response to cobalt is highly concentration dependent, and prolonged ion exposure may compromise biosafety. Therefore, hydrogel incorporation is particularly advantageous for regulating Co^2+^ release and maintaining sustained pro-angiogenic activity within a safe therapeutic window.

Zinc-based MOFs: ZIF-8 is one of the most extensively investigated Zn-based MOFs and consists of tetrahedrally coordinated Zn^2+^ ions linked by 2-methylimidazolate ligands, forming a sodalite-type framework with high physiological stability and acid-responsive degradation (Table S2; Figure S3) [53,55]. Zn-based MOFs exemplify how framework chemistry can couple microenvironmental responsiveness with therapeutic activity. Released Zn^2+^ supports cell proliferation, enzymatic regulation, immune function, collagen synthesis, and tissue remodeling, while also disrupting bacterial membrane integrity, inducing oxidative stress, and inhibiting biofilm formation [53,55]. Their acid-responsive degradation is particularly advantageous for infected wounds, where acidic conditions promote localized Zn^2+^ release. However, pH-triggered ion release should not be equated with direct modulation of resistance-associated signaling pathways, which requires specific mechanistic validation. Integration into hydrogel matrices further enables spatial and temporal control of Zn^2+^ availability, improving therapeutic precision while minimizing excessive ion exposure.

Iron-based MOFs: Representative Fe-based MOFs, including MIL-100(Fe) and MIL-101(Fe), are composed of Fe_3_-oxo clusters coordinated by trimesic acid (BTC), generating mesoporous frameworks with high cargo-loading capacity (Table S2; Figure S3) [57]. Their therapeutic activity is primarily associated with the reversible Fe^3+^/Fe^2+^ redox cycle, which enables modulation of the oxidative microenvironment through both ROS-generating and ROS-scavenging reactions [57]. Consequently, Fe-based MOFs can contribute to antimicrobial activity while simultaneously influencing inflammatory signaling, redox homeostasis, and ferroptosis-related pathways involved in tissue remodeling. These multifunctional properties make iron-based MOFs attractive candidates for hydrogel integration, where controlled degradation and localized ion release may enhance therapeutic efficacy [57]. Nevertheless, because iron can either exacerbate oxidative injury or restore redox balance depending on local physicochemical conditions, defining the therapeutic window remains essential for safe clinical translation.

Magnesium-based MOFs: Magnesium-based MOFs are emerging as regenerative-oriented platforms due to the biological relevance of Mg^2+^ ions in cellular metabolism, signal transduction, endothelial activity, and tissue homeostasis [58]. Compared with Zn-, Cu-, or Ag-based frameworks, Mg-based MOFs are less associated with strong antimicrobial effects and instead provide potential benefits through gradual Mg^2+^ release and modulation of the local regenerative microenvironment. Released Mg^2+^ may contribute to cell adhesion, proliferation, ECM organization, and vascular-related processes [58], although their direct influence on wound-associated signaling pathways remains comparatively underexplored. Their relatively favorable biocompatibility profile makes them attractive candidates for regenerative hydrogel systems [58], where incorporation into polymeric networks can regulate ion diffusion, enhance local retention, and provide a sustained source of Mg^2+^ during tissue repair. Therefore, the therapeutic value of Mg-based MOFs may arise primarily from their ability to complement hydrogel-mediated structural support with biologically relevant ionic cues rather than from intrinsic antimicrobial activity.

Zirconium- and calcium-based MOFs: Zr-based MOFs, particularly UiO-type frameworks, represent a distinct category in hydrogel-based biomedical applications because their primary contribution derives from structural stability, chemical robustness, and controlled molecular delivery rather than direct metal-ion bioactivity. UiO-66 is composed of Zr_6_O_4_(OH)_4_ secondary building units coordinated with terephthalate linkers, generating highly stable frameworks with excellent resistance to aqueous degradation (Table S2; Figure S3) [56]. These characteristics enable efficient encapsulation and protection of therapeutic cargos, including growth factors, anti-inflammatory agents, and antimicrobial molecules, while hydrogel matrices provide a hydrated microenvironment and regulate cargo diffusion. In this context, Zr-based MOFs function as stable nanocarriers within hydrogel networks, expanding the therapeutic versatility of composite systems beyond the biological effects of the metal center itself.

Calcium-containing MOFs provide a complementary regenerative mechanism because Ca^2+^ participates in cellular signaling, adhesion processes, and tissue remodeling [54,57]. Although Ca-based MOFs have been more extensively investigated in bone regeneration than in cutaneous wound repair, their incorporation into hydrogel platforms may offer opportunities to introduce calcium-mediated biological signals while maintaining a supportive three-dimensional matrix. However, similar to other biologically relevant metals, the presence of Ca^2+^ alone does not guarantee regenerative activity, and the therapeutic outcome depends on framework degradation behavior, ion availability, and interactions with the surrounding hydrogel environment [54,57].

### 4.2. From Metal-Ion Release to Molecular Signal Interception

The comparison among metal centers reveals that the therapeutic relevance of MOFs depends not only on which ion is released, but also on where, when, and at what concentration that ion becomes biologically available. Ag^+^ can primarily target microbial viability [15]. Cu^2+^ can simultaneously influence angiogenesis, inflammation, and oxidative stress [55]; Co^2+^ can activate hypoxia-associated angiogenic responses [54]; Zn^2+^ can couple pH-responsive degradation with antimicrobial and regenerative effects [53]; and Fe-based systems can modify redox-dependent processes [57]. These differences indicate that MOFs should be considered programmable sources of biological stimuli rather than passive reservoirs of therapeutic agents.

Nevertheless, the concept of MOFs as “molecular signal interceptors” remains ahead of the mechanistic evidence available for many wound-healing systems. Most studies report changes in bacterial viability, ROS levels, inflammatory markers, angiogenic factors, or wound closure, whereas comparatively few establish a causal relationship between MOF degradation, molecular target engagement, downstream signaling, and the resulting therapeutic phenotype. For example, increased VEGF expression following exposure to a Co-based MOF may be consistent with activation of hypoxia-associated signaling, but VEGF upregulation alone does not establish direct HIF-1α pathway engagement [58,59,60]. Demonstrating such a mechanism would require evidence of HIF-1α stabilization or activation together with downstream target regulation and, ideally, pathway-specific inhibition or genetic validation.

This distinction is particularly important for chronic wounds because the principal pathological processes are interconnected. Microbial persistence can sustain inflammation; excessive inflammation can increase oxidative stress; oxidative stress can impair angiogenesis and cellular proliferation; and biofilm-associated metabolic adaptation can further reduce antimicrobial susceptibility [28,61,62,63]. Consequently, the most promising MOF–hydrogel systems may not be those with the highest activity against an individual target, but those capable of coordinating multiple pathological signals while maintaining a therapeutic window compatible with tissue regeneration.

The integration of MOFs into hydrogels provides an additional level of control over this process. The hydrogel matrix can regulate water and ion transport, increase local retention, limit uncontrolled particle dissemination, and provide complementary biological cues. This creates a materials platform in which MOF composition determines the available molecular stimulus, while the hydrogel controls its spatial presentation and temporal exposure [64,65,66,67,68,69]. Such hierarchical control could be particularly valuable for chronic wounds, where the pathological microenvironment changes dynamically during infection resolution and tissue regeneration.

Despite this potential, mechanistic validation remains a major limitation of the field. Current evidence is largely centered on generalized outcomes such as accelerated wound closure, reduced bacterial load, increased angiogenesis, or decreased inflammatory markers, whereas systematic identification of the molecular targets responsible for these effects remains comparatively scarce [66]. Future studies should therefore move beyond descriptive efficacy assays toward target-validated studies integrating transcriptomics, proteomics, spatial molecular profiling, pathway-specific inhibition, and quantitative ion-release measurements. Establishing these causal relationships will be essential for determining whether MOFs genuinely function as molecular signal interceptors or primarily act through nonspecific physicochemical and antimicrobial effects.

Thus, the future development of MOF–hydrogel systems should prioritize mechanism-guided design rather than simply increasing metal loading or therapeutic cargo content. A clinically relevant platform should establish a quantitative relationship among framework composition, degradation kinetics, ion/cargo availability, molecular target engagement, cellular response, and therapeutic outcome. Such an approach would transform MOFs from multifunctional materials with empirically observed biological effects into rationally engineered interfaces capable of selectively modulating the pathological signaling networks that sustain chronic wounds.

## 5. Hydrogel–MOF Systems for EPI

Bacterial efflux pumps represent one of the major membrane-associated mechanisms contributing to both intrinsic and acquired antimicrobial resistance across diverse bacterial populations [68,70]. These transport systems actively expel structurally diverse compounds from bacterial cells, including antibiotics, antiseptics, and metabolic by-products, thereby reducing intracellular antimicrobial concentrations and contributing to multidrug-resistant phenotypes [70]. In chronic wound biofilms, efflux activity can be influenced by environmental stress, QS, signaling, and prolonged antimicrobial exposure, potentially contributing to bacterial persistence and treatment failure [68,69]. Importantly, efflux pumps also participate in physiological adaptation, virulence, and intercellular signaling, indicating that their biological relevance extends beyond antimicrobial extrusion [71].

Consequently, EPI has emerged as an attractive strategy for restoring antibiotic susceptibility without necessarily increasing antimicrobial dosage. Rather than directly killing microorganisms, EPIs interfere with bacterial transport systems, allowing antibiotics to accumulate intracellularly and potentially recover their antimicrobial activity [66]. This rationale has motivated the development of nanocarrier-based and biomaterial-assisted strategies for localized EPI delivery, including systems designed to co-deliver EPIs and conventional antibiotics [69]. MOFs are particularly attractive for this purpose because of their tunable properties; however, the extent to which MOF-based systems achieve direct EPI, rather than indirect enhancement of antimicrobial activity through membrane disruption, ROS generation, or metal-ion release, remains insufficiently established.

When incorporated into hydrogel matrices, MOFs can further provide localized retention and controlled release of antimicrobial agents and bioactive metal ions. ZIF-8 and copper- or silver-containing MOFs, for example, exhibit intrinsic antimicrobial properties that may complement the activity of conventional antibiotics [72]. MOF-derived metal ions can perturb bacterial membrane integrity, redox homeostasis, and electrochemical gradients, potentially increasing membrane permeability or intracellular antimicrobial accumulation [73]. However, these effects should not be interpreted as direct EPI unless pump activity, transporter expression, or intracellular antimicrobial accumulation is experimentally demonstrated. In this context, hydrogel–MOF composites may be better viewed as multifunctional resistance-modulating platforms in which direct EPI activity, antimicrobial delivery, membrane perturbation, and metal-ion-mediated effects can potentially operate simultaneously [74].

### 5.1. Mechanisms of EPIs

EPIs act through several complementary mechanisms. Some compounds directly bind to efflux proteins and inhibit substrate transport through competitive or non-competitive mechanisms, whereas others interfere with the assembly or function of multiprotein efflux complexes, thereby reducing transport efficiency [74]. Additional mechanisms include dissipation of the proton motive force (PMF), modulation of efflux-related gene expression, and alteration of membrane structure or fluidity, ultimately impairing pump functionality [74]. These mechanisms can increase intracellular antibiotic accumulation and enhance bacterial susceptibility to antimicrobial treatment. However, the mechanistic distinction between direct pump inhibition and nonspecific membrane perturbation remains essential when evaluating resistance-modulating materials.

### 5.2. Hydrogel–MOF Platforms as Resistance-Modulating Systems

Hydrogel–MOF composites provide an opportunity to combine localized antimicrobial delivery with resistance-modulating functions. Their ability to co-deliver antibiotics, EPIs, or other bioactive compounds may maintain therapeutic concentrations at the wound site while reducing systemic exposure [75]. In addition, the MOF component can provide stimuli-responsive degradation and controlled release in response to infection-associated conditions such as acidic pH or elevated ROS levels [76].

MOF-derived metal ions such as Zn^2+^ and Cu^2+^ may further contribute to resistance modulation by altering bacterial membrane integrity, redox homeostasis, and electrochemical gradients [54,77]. These effects may increase membrane permeability, modify intracellular antimicrobial accumulation, or interfere indirectly with energy-dependent transport processes. Nevertheless, metal-ion-mediated membrane disruption should be distinguished from direct inhibition of specific efflux pumps, which requires experimental validation of transporter activity or expression. This distinction is particularly important because an increase in antibiotic susceptibility may arise from several mechanisms that are not necessarily related to efflux inhibition.

Furthermore, the high porosity and tunable surface chemistry of MOFs allow stimuli-responsive release profiles triggered by infection-associated signals such as acidic pH, elevated ROS levels, enzymatic activity, or local temperature changes [78]. Such responsiveness may improve spatial and temporal control of therapeutic exposure while reducing unnecessary drug release and, potentially, associated selective pressure. Nevertheless, whether these systems reduce resistance selection in vivo remains insufficiently established and should not be inferred solely from controlled-release behavior.

### 5.3. Stimuli-Responsive Mechanisms and Antibacterial Performance

Beyond direct efflux inhibition, hydrogel–MOF systems can modulate bacterial resistance through oxidative, photothermal, photodynamic, and membrane-associated mechanisms. Several MOFs can generate ROS or catalyze oxidative reactions that damage bacterial membranes, proteins, nucleic acids, and biofilm components [41]. These effects may compromise membrane integrity and increase antibiotic penetration or intracellular accumulation. However, increased intracellular antibiotic levels resulting from membrane damage should not by themselves be interpreted as evidence of EPI.

Stimuli-responsive hydrogel–MOF composites can further enhance these effects through on-demand release mechanisms. Systems activated by pH, enzymes, temperature, or near-infrared (NIR) irradiation can undergo matrix degradation or structural transitions that facilitate localized release of encapsulated antibiotics or other therapeutic cargoes within infected tissues [72]. NIR-responsive platforms may additionally generate localized photothermal effects that increase membrane permeability and alter bacterial protein function, thereby enhancing intracellular antimicrobial accumulation [72]. When combined with photothermal or photodynamic therapy, these systems can generate localized hyperthermia and oxidative stress that further compromise bacterial membranes and biofilm integrity. Such effects may contribute to increased antimicrobial susceptibility, but they should be distinguished from direct inhibition of defined efflux transporters unless specific pump activity has been experimentally assessed.

Recent studies involving ZIF-8 nanoparticles, copper-based MOFs, and related hybrid systems have reported enhanced intracellular drug accumulation, ROS-mediated membrane disruption, antibiofilm activity, and reductions in minimum inhibitory concentration (MIC) values against resistant pathogens such as *Staphylococcus aureus* and *Escherichia coli* [72]. These findings demonstrate the potential of MOF-based systems to enhance antimicrobial efficacy and modulate phenotypic resistance. However, reduced MIC values or increased bacterial killing do not, by themselves, establish direct EPI because similar outcomes may result from increased drug delivery, membrane permeabilization, oxidative damage, or other physicochemical mechanisms.

### 5.4. Toward Resistance-Modulating Wound Interfaces

The potential significance of hydrogel–MOF systems extends beyond conventional antimicrobial activity because these platforms can integrate localized drug delivery, membrane modulation, ROS-mediated effects, and, potentially, EPI. By promoting intracellular antimicrobial accumulation and altering bacterial membrane or metabolic processes, these materials may complement conventional antibiotics without relying exclusively on increased antimicrobial dosage. However, current evidence should be carefully distinguished between direct and indirect resistance modulation.

Many reported MOF-based systems primarily rely on controlled antimicrobial release, photothermal or photodynamic activity, ROS generation, or metal-ion-mediated membrane disruption rather than experimentally validated inhibition of a specific efflux system [75]. Consequently, reduced MIC values, increased antibiotic accumulation, or improved bacterial killing should not be considered sufficient evidence of EPI. Direct evidence would require complementary assessment of efflux activity, intracellular antibiotic accumulation, transporter expression or function, and, where possible, genetic or pharmacological validation of the targeted pump.

**Figure 3:**
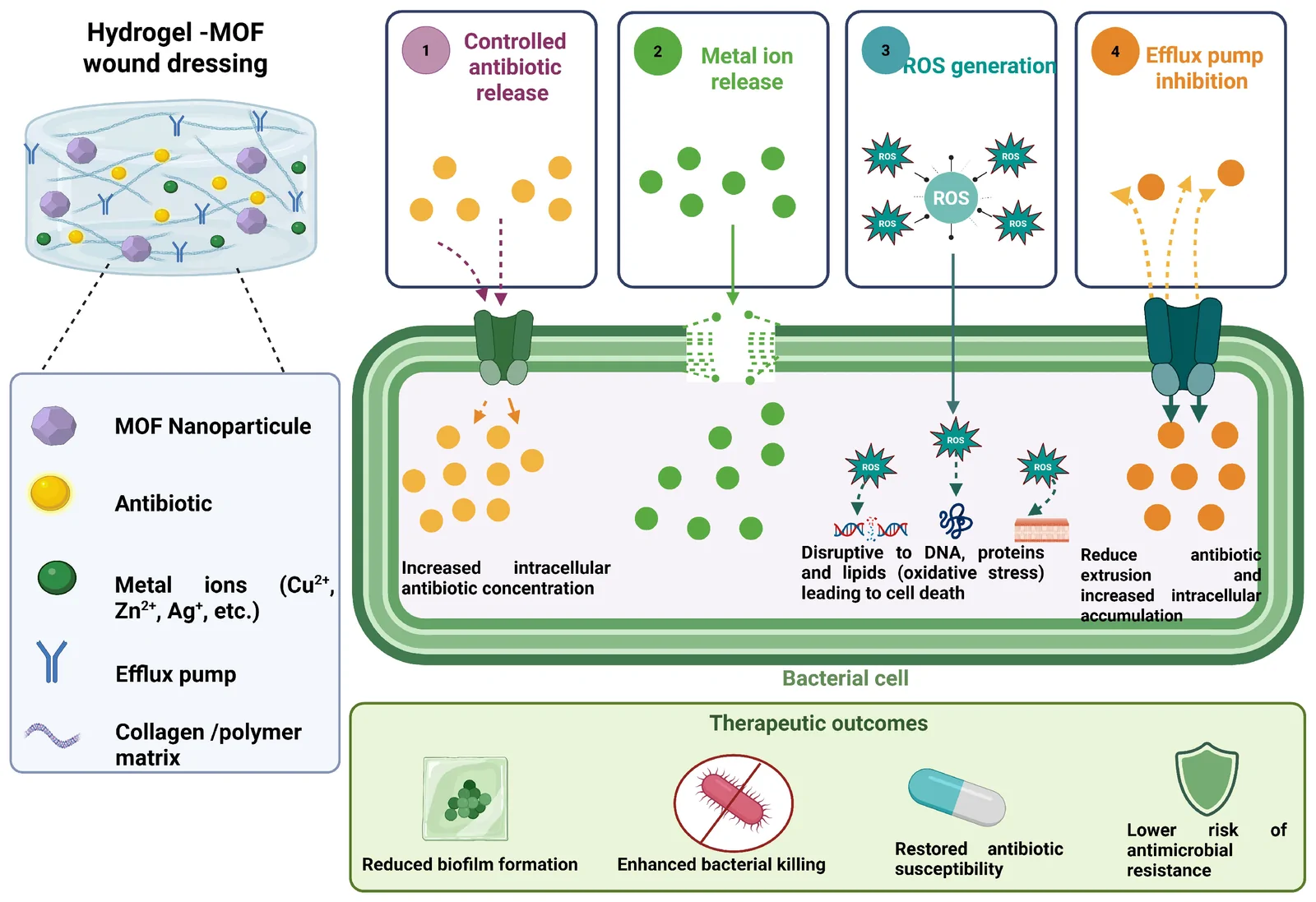
Schematic representation of the main mechanisms involved in efflux pump inhibition by hydrogel–MOF wound dressings, including antibiotic and efflux pump inhibitor delivery, metal ion release, ROS generation, and stimuli-responsive therapeutic release. Created in BioRender. Rizo, J. (2026) https://BioRender.com/xvwq0ur (accessed on 31 July 2026).

This distinction represents an important research gap for hydrogel–MOF systems. Current research has extensively explored MOFs and related nanocarriers for antimicrobial delivery and resistance-associated phenotypes [69,74], whereas the rational engineering of MOF–hydrogel interfaces specifically for direct EPI remains comparatively underexplored. Future studies should therefore prioritize the identification of specific efflux targets, quantitative determination of EPI loading and release, evaluation of antibiotic–EPI synergy, and mechanistic discrimination between direct pump inhibition and indirect membrane, redox, or metabolic perturbation. In parallel, the metabolic costs of resistance modulation, long-term material safety, dose optimization, and scalability should be considered to determine whether these systems may provide sustained resistance-modulating effects under clinically relevant wound conditions. Representative hydrogel–MOF systems and their proposed resistance-modulating mechanisms are summarized in Table 3. The synergistic mechanisms involved—including co-delivery of antibiotics and EPIs, metal-ion release, ROS generation, membrane disruption, and stimuli-responsive therapeutic release—are illustrated in Figure 3.

## 6. EPS Disruption and Biofilm Destabilization Mechanisms

The persistence of biofilms in chronic wounds is determined not only by the intrinsic resistance of bacterial cells but also by the protective functions of the EPS matrix. This highly hydrated and dynamic structure, composed primarily of polysaccharides, proteins, lipids, and extracellular DNA (eDNA), regulates bacterial adhesion, nutrient transport, intercellular communication, and adaptation to environmental stressors [83]. Beyond serving as a structural scaffold, the EPS matrix acts as a viscoelastic barrier that can restrict antimicrobial penetration, attenuate host immune responses, and generate physicochemical gradients that favor bacterial survival under adverse conditions [84]. In chronic wounds, the EPS contributes to heterogeneous microenvironments characterized by oxygen depletion, nutrient gradients, pH fluctuations, and redox imbalance, which collectively promote bacterial persistence and antimicrobial tolerance [85,86]. Consequently, therapeutic strategies focused exclusively on bacterial eradication may be insufficient when the structural and physicochemical properties of the biofilm matrix remain largely intact. Increasing evidence therefore supports approaches that target both bacterial cells and the extracellular architecture that sustains collective protection. In this context, bioactive hydrogel–MOF composites have been investigated as multifunctional platforms capable of modifying biofilm structure through complementary physicochemical mechanisms, including osmotic, electrostatic, ionic, and oxidative effects. Such mechanisms may progressively destabilize the EPS matrix and improve the accessibility of therapeutic agents to embedded bacterial populations. Representative hydrogel–MOF systems associated with EPS disruption are summarized in Table 4.

### 6.1. Osmotic and Electrostatic Destabilization of the EPS

The initial stages of biofilm disruption may occur at the hydrogel–EPS interface, where osmotic and physicochemical interactions can influence matrix organization. Because highly hydrophilic hydrogels can undergo substantial swelling upon hydration, differences in water activity and polymer–water interactions may alter the local hydration state and mechanical properties of the surrounding biofilm matrix [91]. Under specific material and environmental conditions, these interactions may contribute to changes in EPS viscoelasticity and facilitate subsequent physical or chemical disruption [84,91]. However, the magnitude and direction of these effects depend strongly on hydrogel composition, crosslinking density, swelling behavior, and the physicochemical properties of the biofilm.

Electrostatic interactions may provide an additional mechanism for EPS destabilization. Many extracellular polysaccharides and eDNA carry negative charges under physiological conditions and contribute to the organization and cohesion of the biofilm matrix [92]. Charged biopolymers or functionalized hydrogel networks can interact with these components and modify intermolecular associations, including electrostatic interactions, hydrogen bonding, and hydrophobic interactions that contribute to matrix cohesion [93]. Such interactions may weaken matrix organization and facilitate biofilm dispersion or increase bacterial exposure to antimicrobial agents and host immune factors [94]. Nevertheless, the extent of electrostatic disruption is dependent on polymer charge density, ionic strength, pH, and the molecular composition of the EPS.

### 6.2. MOF-Mediated Ionic Destabilization of Biofilms

The incorporation of MOFs into hydrogel matrices introduces an additional mechanism for biofilm modulation through localized metal-ion release. Depending on their composition, metal ions such as Zn^2+^, Cu^2+^, and Fe^2+^ can interact with functional groups present in polysaccharides, proteins, and eDNA and thereby modify molecular interactions within the EPS network. Biofilm stability can also depend on divalent cations such as Ca^2+^ and Mg^2+^, which contribute to the coordination and crosslinking of negatively charged extracellular polymers and eDNA [95,96]. Consequently, perturbation of these ionic interactions may alter matrix organization and mechanical stability.

The extent to which MOFs participate in ion exchange, competitive coordination, or metal-mediated reorganization of the EPS depends on the coordination chemistry, metal center, ligand environment, SBU, and degradation behavior of the specific framework. Where such interactions occur, localized metal-ion release may modify EPS crosslinking, increase matrix permeability, and facilitate diffusion of therapeutic molecules into previously protected biofilm regions [75,94]. Thus, hydrogel–MOF systems may contribute to biofilm destabilization not only through direct antimicrobial effects but also through physicochemical modification of the ECM.

### 6.3. Oxidative Degradation of the EPS

In addition to ionic effects, selected MOFs can function as catalytic or photocatalytic platforms capable of generating ROS within the biofilm microenvironment [97]. Depending on the metal center, ligand structure, redox properties, and external stimuli, MOFs can promote localized oxidative reactions that modify extracellular biomolecules. When incorporated into hydrogel matrices, the spatial confinement of these materials may provide additional control over their localization and release, although the extent to which such confinement limits collateral oxidative damage to host tissue remains system-dependent [98].

Among the different EPS components, eDNA represents an important potential target because it contributes to matrix stabilization and can facilitate horizontal gene exchange within bacterial communities [99]. ROS generated through redox-active metal centers such as Fe- and Cu-containing systems may promote oxidative modification or degradation of extracellular macromolecules, including nucleic acids, polysaccharides, and proteins [100]. Such reactions can alter matrix viscosity, organization of water channels, and the physicochemical environment surrounding bacterial communities [101]. Oxidative modification of structural EPS components may consequently weaken matrix cohesion and facilitate biofilm dispersion [101].

Beyond direct matrix modification, oxidative processes may increase biofilm permeability and facilitate antimicrobial diffusion while altering the nutrient and oxygen gradients that contribute to bacterial persistence [102]. Thus, hydrogel–MOF systems may affect biofilm susceptibility through multiple interconnected mechanisms rather than through bacterial killing alone. Overall, osmotic, electrostatic, ionic, and oxidative mechanisms represent complementary strategies for modifying the extracellular architecture of biofilms. Their therapeutic relevance, however, depends on the extent, spatial distribution, and temporal kinetics of matrix disruption relative to antimicrobial delivery and host-tissue exposure.

**Figure 4:**
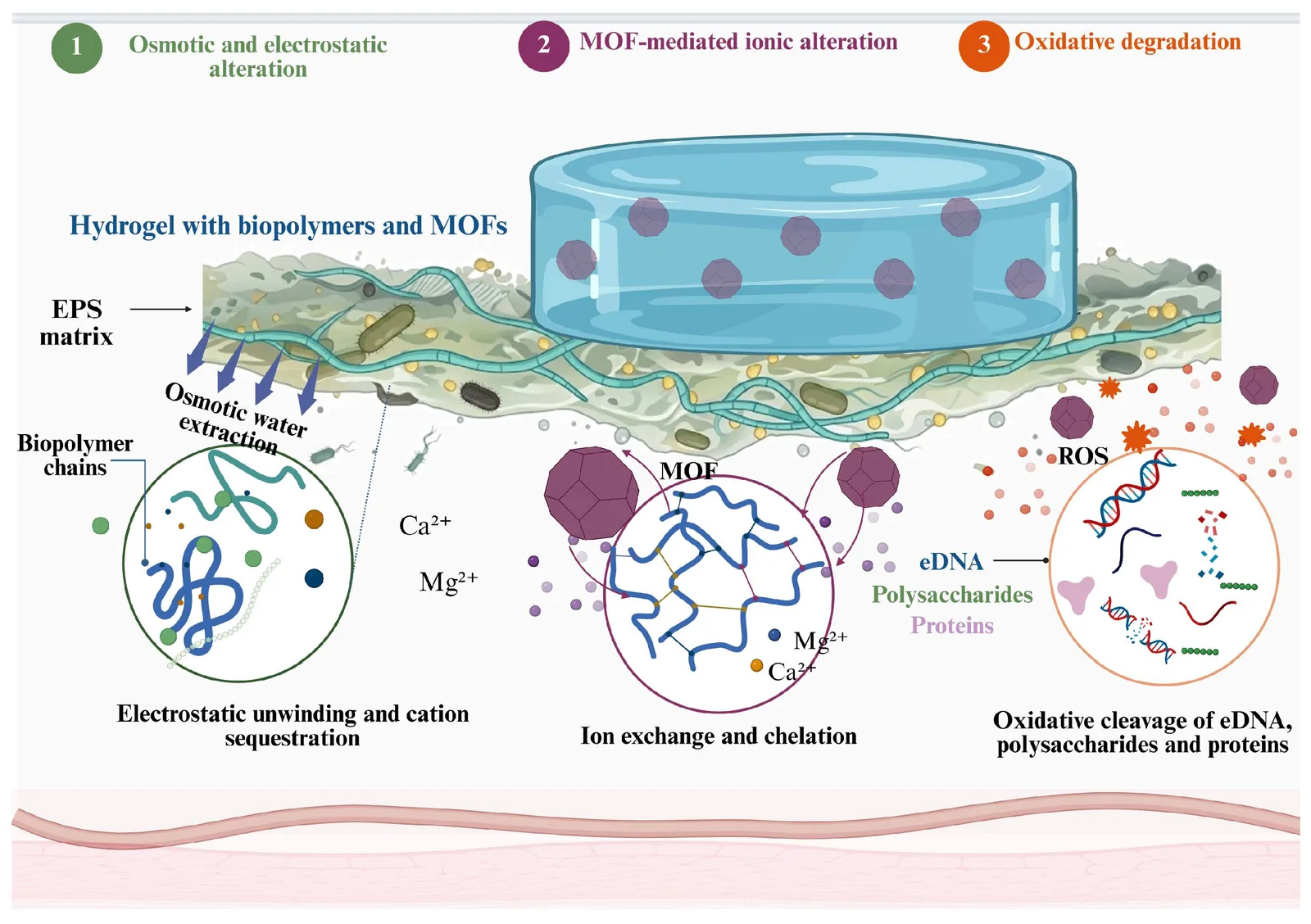
Mechanisms of EPS destabilization mediated by the bioactive hydrogel–MOF system. Created in BioRender. Rizo, J. (2026) https://BioRender.com/mh1azsi (accessed on 31 July 2026).

This distinction is particularly relevant for chronic wound applications, where complete biofilm destabilization cannot be inferred from reductions in bacterial viability alone. While the structural complexity of EPS and the potential of MOF-based strategies to modify biofilm architecture are increasingly recognized, important mechanistic gaps remain regarding the spatial and temporal kinetics required to achieve functionally meaningful matrix destabilization [84]. Current studies often rely on simplified in vitro biofilm models that do not fully reproduce the heterogeneous oxygen, nutrient, fluid, mechanical, and immune conditions encountered in chronic wounds. In particular, the extent to which hydrogel–MOF systems may overcome matrix-mediated transport barriers, eDNA-associated protection, and nutrient- and oxygen-limited microenvironments remains insufficiently characterized. Future studies should therefore integrate quantitative measurements of EPS composition, viscoelasticity, permeability, metal-ion transport, ROS distribution, and antimicrobial penetration under physiologically relevant biofilm conditions. The principal mechanisms proposed to contribute to EPS disruption by hydrogel–MOF systems are summarized in Figure 4.

Importantly, increased antibiofilm activity should not be interpreted as direct evidence of EPS disruption. Reduced viable bacterial counts, inhibition zones, or decreases in total biofilm biomass may result from direct bactericidal activity of released metal ions, antibiotics, ROS, or photothermal effects without substantial modification of the ECM. Demonstrating an EPS-directed mechanism therefore requires complementary structural, biochemical, and functional evidence. Quantification of extracellular polysaccharides, proteins, and eDNA should ideally be combined with three-dimensional imaging, rheological or mechanical characterization of the biofilm matrix, and measurements of antimicrobial penetration. Such analyses would allow researchers to distinguish between direct bacterial killing and genuine matrix destabilization. More importantly, demonstrating increased antimicrobial penetration following EPS disruption would establish a mechanistic link between matrix modification and improved therapeutic efficacy. This distinction is particularly relevant for hydrogel–MOF systems because their antimicrobial performance may arise from multiple simultaneous mechanisms, including metal-ion toxicity, ROS generation, photothermal effects, and controlled antibiotic release. Future studies should therefore incorporate mechanism-specific controls capable of separating direct bactericidal effects from EPS-directed biofilm destabilization.

## 7. Modulating HGT in Wound Biofilms

Chronic wound biofilms function as dynamic genetic reservoirs in which the EPS matrix extends beyond its structural role and can contribute to the persistence and dissemination of AMR determinants [103]. The high bacterial density, prolonged cell-to-cell contact, and spatial confinement characteristic of biofilm communities can facilitate HGT, particularly through conjugation and natural transformation [104]. As an important driver of microbial evolution and adaptation, HGT enables the dissemination of resistance determinants through mobile genetic elements (MGEs), including plasmids, transposons, integrons, and bacteriophages [105].

Compared with planktonic populations, bacteria embedded within biofilms may exhibit enhanced opportunities for genetic exchange because of the close physical proximity and prolonged interactions maintained within the EPS matrix [106]. In these environments, eDNA performs a dual function by contributing to biofilm structural stability while potentially serving as a reservoir of antibiotic resistance genes (ARGs) available for horizontal acquisition [104]. Consequently, chronic wound biofilms can function as environments that favor the persistence and dissemination of resistance-associated genetic elements, particularly under conditions of antimicrobial exposure, oxidative stress, and sustained microbial competition [107].

The efficiency of HGT is further influenced by the heterogeneous physicochemical conditions characteristic of chronic wound biofilms. Hypoxia, acidic pH, nutrient gradients, and redox imbalance can stimulate adaptive bacterial responses, stress-induced competence, and resistance-associated gene expression [104]. QS-mediated communication networks additionally coordinate collective behaviors involved in biofilm maturation, virulence regulation, metabolic adaptation, and interspecies interactions, thereby contributing to microbial persistence within polymicrobial communities [108]. The EPS matrix can also protect extracellular nucleic acids and mobile genetic elements from degradation, while physiological heterogeneity promotes the emergence of metabolically distinct bacterial subpopulations, including dormant and persister cells with increased tolerance to antimicrobial stress [104]. Collectively, these factors indicate that AMR dissemination within chronic wound biofilms depends not only on bacterial abundance but also on the physicochemical and ecological conditions that influence genetic exchange and adaptive evolution, as summarized in Table 5.

Importantly, the intervention strategies summarized in Table 5 should be regarded primarily as mechanistic opportunities rather than established anti-HGT effects, because most available studies evaluate bacterial viability, biofilm biomass, or physicochemical properties rather than directly quantifying genetic-transfer frequencies.

These observations have stimulated interest in anti-evolutionary therapeutic strategies that target resistance dissemination pathways rather than relying exclusively on bactericidal activity. Within this framework, bioactive hydrogel–MOF composites may provide multifunctional wound interfaces capable of modifying microenvironmental conditions associated with HGT. Depending on their physicochemical properties, hydrogel matrices may influence bacterial mobility, cell–cell interactions, molecular diffusion, and the spatial distribution of extracellular nucleic acids, potentially modifying conditions that favor genetic exchange [104]. In parallel, MOFs may influence microbial communities through controlled ion release, catalytic redox activity, and, for selected systems, modulation of bacterial signaling pathways [109].

Stimuli-responsive MOFs are particularly relevant because pathological conditions commonly encountered in chronic wounds, including acidic pH and oxidative stress, can be exploited to regulate ion or cargo release. Such responsiveness may enhance local biofilm disruption while limiting unnecessary exposure of surrounding tissue [106]. Likewise, Zn-based MOFs have been associated with alterations in bacterial aggregation, biofilm organization, and signaling processes that may modify microenvironments favorable to genetic exchange and increase bacterial susceptibility to antimicrobial or immune-mediated clearance [110]. However, these effects should not be interpreted as direct evidence of HGT inhibition unless genetic-transfer frequency or the mobilization of resistance determinants is experimentally quantified.

**Figure 5:**
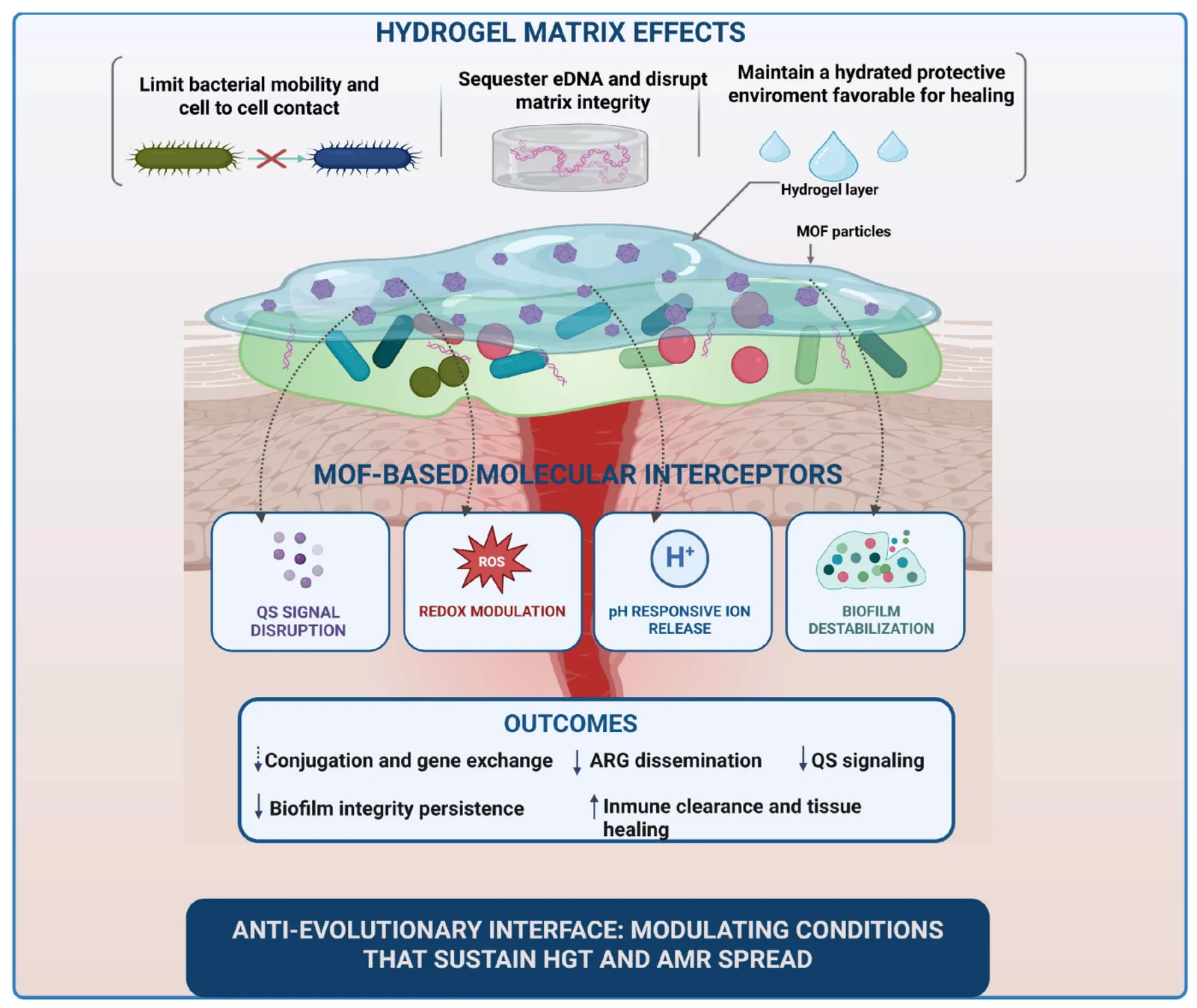
Hydrogel–MOF interfaces may disrupt HGT-associated pathways through eDNA sequestration, bacterial communication interference, redox modulation, pH-responsive ion release, and biofilm destabilization. Created in BioRender. Rizo, J. (2026) https://BioRender.com/3o6y546 (accessed on 31 July 2026).

Rather than functioning solely as antimicrobial platforms, adaptive hydrogel–MOF composites may therefore represent a potential class of resistance-modulating wound interfaces capable of influencing the physicochemical, ecological, and signaling conditions associated with AMR dissemination. By simultaneously modifying microbial communication, biofilm organization, and the local availability of resistance-associated genetic elements, these systems may potentially interfere with processes that sustain resistance within chronic wound ecosystems. The principal pathways through which hydrogel–MOF systems may influence HGT and resistance dissemination are illustrated in Figure 5. Future hydrogel–MOF wound dressings may thus evolve beyond passive antimicrobial barriers toward adaptive anti-resistance platforms capable of dynamically regulating microbial communication, genetic exchange, and biofilm-associated evolutionary processes.

Importantly, the distinction between biofilm disruption and direct HGT inhibition remains insufficiently addressed. Current therapeutic approaches predominantly focus on reducing bacterial burden and disrupting biofilm structure, whereas comparatively fewer strategies directly target the molecular processes governing genetic exchange [111]. Critical gaps therefore include the development of HGT-specific therapeutic strategies, quantitative monitoring of gene-transfer events within physiologically relevant wound models, and evaluation of whether antimicrobial interventions themselves alter the selective and ecological conditions that influence resistance evolution [111]. For hydrogel–MOF systems, demonstrating a genuine anti-HGT effect will require measurements that extend beyond bacterial viability or biofilm biomass and directly quantify conjugation, transformation, transduction, plasmid transfer, or resistance-gene mobilization under relevant wound conditions.

## 8. Rational Design of Resistance-Modulating Bioactive MOF–Hydrogel Wound Dressings

Resistance-modulating wound interfaces represent an emerging class of multifunctional biomaterial platforms designed to regulate the biological processes that contribute to antimicrobial resistance development within chronic wound environments. Unlike conventional antibacterial dressings, which primarily rely on direct microbial elimination, these interfaces aim to interfere with resistance-associated phenomena, including biofilm persistence, EPS stabilization, efflux-mediated tolerance, and HGT, while simultaneously restoring a favorable regenerative microenvironment. In this context, hydrogel–MOF systems provide a unique design opportunity because the hydrogel component can regulate hydration, molecular transport, cellular interactions, and microenvironmental conditions, whereas MOFs can introduce catalytic activity, ion-mediated regulation, and controlled delivery functions. Therefore, resistance modulation emerges not from a single antibacterial mechanism but from the coordinated interaction between material architecture, physicochemical responsiveness, and biological regulation.

The previous sections described the mechanisms underlying AMR and the multiscale interactions of hydrogel–MOF systems within chronic wound environments. However, understanding these individual mechanisms represents only the first step toward developing clinically relevant wound dressings. The design of next-generation hydrogel–MOF platforms requires the integration of structural, physicochemical, and biological parameters within multifunctional systems capable of responding to the wound microenvironment, pathogenic microorganisms, and complementary therapeutic agents. Their therapeutic performance will ultimately depend on the rational combination of modular functionalities that can be spatially and temporally adapted to the evolving requirements of chronic wounds [21]. One important design consideration is the architecture of semi-interpenetrating polymer network (semi-IPN) systems. Network pore size and connectivity influence mass transport, oxygen diffusion, cellular infiltration, fluid exchange, and the incorporation and spatial distribution of MOFs within the polymer matrix. Depending on the intended application and interfacial chemistry, MOFs may be chemically integrated into polymer networks or physically incorporated through non-covalent interactions, with each strategy providing different effects on structural stability, particle retention, and release behavior [112]. Importantly, the hydrogel phase should not be considered merely as a passive support for MOF nanoparticles. Instead, polymer–MOF interactions determine particle dispersion, accessibility of active sites, degradation kinetics, and the spatial distribution of therapeutic signals. Conversely, MOF incorporation can modify hydrogel properties by influencing network organization, mechanical behavior, and biochemical interactions with the wound microenvironment. Pore architecture must therefore be optimized according to the specific biological and transport requirements of the target wound. Pore dimensions have frequently been investigated in tissue-engineering and wound-healing scaffolds because they can support cellular migration, fluid transport, and tissue infiltration [112]. However, no universal pore size is optimal for all wound types, and the appropriate architecture depends on polymer composition, crosslinking density, wound geometry, cell population, and therapeutic objective. Thus, pore size should be considered as a tunable design parameter rather than an independent predictor of therapeutic performance. The principal design considerations for engineering resistance-modulating hydrogel–MOF wound dressings are summarized in Figure S4.

Another critical parameter is responsiveness to wound-associated stimuli. Chronic wounds exhibit dynamic variations in pH, ROS levels, protease activity, oxygen availability, and inflammatory signaling. Smart hydrogel–MOF systems can exploit these pathological cues to regulate the release of antimicrobial, anti-inflammatory, or regenerative agents. pH-responsive MOFs, redox-sensitive linkages, enzyme-responsive polymer networks, and other stimulus-responsive architectures may therefore provide mechanisms for spatially and temporally controlled therapeutic delivery [112]. However, stimulus responsiveness should not be considered sufficient evidence of therapeutic selectivity. The magnitude, kinetics, reversibility, and biological consequences of the response must be characterized under conditions that reproduce the relevant wound microenvironment. Such validation is particularly important for chronic wounds, where pathological conditions may vary considerably across spatially distinct regions and throughout the healing process.

Beyond conventional antimicrobial activity, advanced hydrogel–MOF dressings are being explored as resistance-modulating interfaces by incorporating functionalities capable of interfering with bacterial persistence and adaptation mechanisms. These functionalities include EPIs, biofilm-disrupting agents, and strategies designed to interfere with resistance-gene dissemination [19]. These components provide an opportunity to target bacterial adaptation mechanisms in parallel with pathogen elimination. However, the incorporation of such functionalities should not be equated with demonstrated resistance reversal. As discussed in previous sections, direct evidence of EPI, EPS disruption, or HGT suppression requires mechanism-specific experimental validation. Consequently, future hydrogel–MOF platforms should be designed to combine antimicrobial activity with independently validated resistance-modulating mechanisms rather than assuming that improved bacterial killing represents reversal of AMR.

The physicochemical properties of semi-IPN systems ultimately determine their transport, mechanical, and therapeutic behavior. These characteristics influence the diffusion and release of metal ions, antimicrobial compounds, signaling molecules, and other therapeutic agents, while also determining the ability of the dressing to conform to irregular wound geometries and maintain an appropriate moisture balance [111]. Among these properties, crosslinking density plays an important role because it affects network mesh size, swelling, mechanical strength, degradation, and molecular diffusion. Increasing crosslinking density generally restricts water uptake and molecular mobility while improving structural stability; conversely, lower crosslinking densities typically increase swelling and fluid uptake [113]. However, the relationship between crosslinking and release kinetics is not universal because release can be governed simultaneously by diffusion, matrix degradation, drug–polymer interactions, MOF degradation, and ion exchange [114]. Rational design should therefore optimize these parameters collectively rather than treating crosslinking density as an isolated determinant of release.

Biocompatibility represents another fundamental requirement for the clinical translation of hydrogel–MOF dressings. Although many polymers used in semi-IPN systems originate from natural or bioinspired materials, the biological safety of the final composite depends on the chemical composition of the hydrogel, MOF stability, degradation products, and local concentrations of released metal ions [112]. Excessive or poorly controlled ion release may induce cytotoxicity, alter cellular proliferation, increase oxidative stress, or interfere with tissue regeneration. Accordingly, evaluation should extend beyond antibacterial efficacy to include degradation kinetics, ion release profiles, inflammatory responses, cytocompatibility, and tissue remodeling under relevant exposure conditions [115,116,117,118].

Finally, the biodegradation profile of hydrogel–MOF dressings should be matched to the temporal requirements of wound repair. An appropriate system should maintain sufficient structural integrity and therapeutic functionality during the relevant stages of healing while progressively degrading into products that can be safely metabolized, cleared, or removed [119]. Controlled degradation may reduce the frequency of dressing replacement and minimize secondary tissue trauma during removal. Importantly, degradation should be evaluated together with MOF disassembly and metal-ion release because these processes may occur at different rates and therefore produce temporally distinct biological exposures.

Collectively, these considerations define hydrogel–MOF systems as resistance-modulating wound interfaces rather than conventional antimicrobial dressings. Their therapeutic potential relies on the coordinated regulation of microbial behavior, resistance-associated processes, and host tissue responses through the integration of material design and biological functionality. Table S3 summarizes the principal design parameters governing therapeutic performance, resistance modulation, and tissue compatibility.

Despite the potential of resistance-modulating bioactive MOF wound dressings, substantial challenges remain in translating these multifunctional systems into predictable therapeutic platforms. A major challenge is achieving localized and stimuli-responsive release while simultaneously controlling MOF degradation, metal-ion exposure, and therapeutic cargo availability within the hydrogel matrix [114]. The biological consequences of metal-ion release also require careful consideration because the same ions that provide antimicrobial or regenerative effects may become cytotoxic at higher local concentrations [120]. Moreover, systematic comparisons of synthesis procedures, composition, particle distribution, degradation kinetics, release profiles, and biological performance are still required to improve reproducibility and scalability [112]. Of particular importance, future studies should establish quantitative relationships among MOF composition, hydrogel architecture, degradation, ion and cargo release, molecular target engagement, resistance-modulating activity, and tissue response. Such mechanism-guided design will be essential for transforming hydrogel–MOF composites from multifunctional experimental materials into predictable and clinically translatable resistance-modulating wound interfaces. To integrate the structural, physicochemical, and biological design considerations discussed above, representative hydrogel–MOF systems investigated for wound management are comparatively evaluated in Table 6. Particular attention is given to their demonstrated biological functions and to the extent to which these properties may contribute to AMR modulation. Importantly, antibacterial or antibiofilm activity is not considered equivalent to direct interference with resistance mechanisms. Therefore, the comparison distinguishes experimentally demonstrated antimicrobial effects from indirect or proposed effects on resistance-associated processes, including biofilm persistence, microbial adaptation, and the local wound microenvironment. This distinction highlights both the multifunctional potential of hydrogel–MOF systems and the current mechanistic gap between antimicrobial efficacy and experimentally validated resistance modulation.

## 9. Future Perspectives

Despite substantial progress in the development of hydrogel–MOF wound dressings, significant scientific, technological, and regulatory challenges remain before these multifunctional systems can be considered for clinical translation [63,97,124,125]. Future advances in this field will require a hierarchical framework integrating fundamental mechanistic understanding, material engineering optimization, cross-disciplinary innovative strategies, and systematic clinical translation assessment. Such an approach is essential to transform hydrogel–MOF composites from multifunctional experimental materials into predictable resistance-modulating wound interfaces.

### 9.1. Fundamental Mechanistic Exploration

A major challenge for the future development of resistance-modulating hydrogel–MOF systems is establishing quantitative relationships between material characteristics and biological outcomes. Although hydrogel–MOF composites have demonstrated antimicrobial and antibiofilm effects, reductions in bacterial viability, biofilm biomass, or microbial burden do not necessarily demonstrate direct modulation of antimicrobial resistance mechanisms. Future investigations should therefore move beyond empirical multifunctionality and incorporate mechanism-specific analyses to determine whether these systems genuinely interfere with resistance-associated biological processes.

In particular, future studies should clarify how polymer–MOF interactions regulate therapeutic responses within chronic wound environments. The coupling between hydrogel architecture, MOF degradation, metal-ion availability, cargo release, molecular target engagement, bacterial adaptation, and tissue regeneration remains insufficiently understood. Mechanistic validation should include quantitative evaluation of efflux activity, intracellular antibiotic accumulation, expression and activity of resistance-associated pathways, extracellular polymeric substance disruption, conjugation or transformation assays, and longitudinal measurements of resistance evolution [19,74,111]. These approaches will be essential to distinguish genuine resistance modulation from conventional antimicrobial effects and to establish whether hydrogel–MOF systems can influence bacterial adaptation rather than only reduce microbial viability.

### 9.2. Material Engineering Optimization

From a materials engineering perspective, future efforts should focus on controlling the hydrogel–MOF interface to achieve predictable structure–property–performance relationships. Manufacturing reproducibility remains one of the major challenges for the translation of these hybrid systems [114]. Reproducible MOF synthesis, homogeneous incorporation and spatial distribution within hydrogel matrices, and maintenance of batch-to-batch consistency are essential requirements for scalable production. These challenges may become particularly relevant when naturally derived biopolymers are employed because their physicochemical characteristics can vary depending on biological source, extraction procedures, purification strategies, and processing conditions [112]. Standardized characterization protocols will therefore be required to establish reliable relationships between composite architecture, biological functionality, and therapeutic performance.

Long-term structural stability represents another critical engineering challenge for hydrogel–MOF wound interfaces. Stable retention of MOF components within polymer networks, synchronization between hydrogel degradation and wound-healing progression, and preservation of mechanical integrity under physiological conditions are important determinants of therapeutic efficacy [112]. Premature framework degradation or uncontrolled particle release may alter metal-ion and cargo exposure, whereas excessive structural persistence may interfere with tissue remodeling or dressing removal. Therefore, future studies should evaluate hydrogel degradation, MOF disassembly, particle retention, and metal-ion release as interconnected processes rather than as independent material properties.

Advanced manufacturing strategies may provide additional opportunities to overcome current limitations in hydrogel–MOF fabrication and functional integration. Electrospinning, three-dimensional bioprinting, microfluidics-assisted fabrication, and hierarchical assembly approaches can provide improved control over architecture, porosity, mechanical properties, MOF distribution, and spatial localization of therapeutic components [112,126,127]. These strategies may enable the development of wound interfaces with compartmentalized functions, where antimicrobial, antibiofilm, resistance-modulating, and regenerative activities are spatially and temporally coordinated according to wound requirements. However, increasing structural complexity should be accompanied by rigorous quality-control strategies to ensure reproducibility, scalability, and consistent biological performance.

Beyond architectural optimization, future engineering efforts should focus on controlling the dynamic interactions between hydrogel networks and MOF components during therapeutic operation. The relationship between crosslinking density, swelling behavior, molecular diffusion, MOF retention, and therapeutic release should be systematically optimized rather than evaluated independently. Establishing these correlations will be essential to achieve controlled exposure of metal ions, antimicrobial compounds, and signaling molecules while maintaining adequate mechanical integrity and biological compatibility [114].

### 9.3. Cross-Disciplinary Innovative Designs

Future generations of resistance-modulating hydrogel–MOF wound dressings will require integration with advanced biological characterization strategies and interdisciplinary approaches capable of capturing the complexity of chronic wound ecosystems. Chronic wounds are dynamic environments characterized by microbial community interactions, resistance-gene dissemination, inflammatory signaling, and continuous changes in host–microbe relationships. Therefore, molecular and spatial biology approaches, including metagenomics, transcriptomics, proteomics, spatial imaging, single-cell analysis, and longitudinal microbiome profiling, may provide essential information regarding microbial adaptation, resistance-associated pathways, QS networks, metabolic heterogeneity, and tissue responses [120].

Integration of these biological datasets with quantitative measurements of MOF degradation, metal-ion availability, therapeutic release profiles, and tissue remodeling responses may enable identification of molecular signatures associated with treatment success or failure. Such approaches could facilitate the development of adaptive hydrogel–MOF platforms capable of responding to specific wound conditions rather than applying uniform therapeutic strategies.

Furthermore, future designs may benefit from combining advanced material fabrication with predictive computational approaches. Data-driven strategies integrating material composition, structural parameters, biological responses, and therapeutic outcomes could accelerate the identification of optimized hydrogel–MOF configurations and reduce the empirical nature of current development approaches.

### 9.4. Clinical Translation Assessment of Hydrogel–MOF Wound Dressings

Despite their multifunctional potential, the clinical translation of resistance-modulating hydrogel–MOF systems requires comprehensive evaluation of safety, reproducibility, and therapeutic predictability. Although many hydrogel-forming polymers exhibit favorable biocompatibility, the biological consequences of prolonged exposure to MOFs, released metal ions, degradation products, and residual synthesis components require systematic investigation [126]. In particular, the therapeutic window between antimicrobial or resistance-modulating activity and potential host-cell toxicity must be quantitatively established.

Future evaluations should extend beyond short-term antibacterial assays and include acute and chronic cytotoxicity, inflammatory and immunological responses, degradation products, local metal-ion accumulation, biodistribution when relevant, and tissue remodeling under clinically representative exposure conditions [127]. These assessments should also consider the intended duration of use and repeated exposure scenarios associated with chronic wound management.

Another important challenge is the establishment of standardized evaluation protocols that allow meaningful comparison among different hydrogel–MOF systems. Future studies should integrate manufacturing parameters, composite composition, particle distribution, degradation kinetics, release profiles, molecular target engagement, resistance-modulating activity, and tissue responses into unified assessment frameworks [112]. Such standardization will be critical for improving reproducibility and facilitating regulatory evaluation.

Ultimately, the successful translation of hydrogel–MOF wound dressings will depend on shifting from empirical multifunctionality toward mechanism-guided and quantitatively validated design. Future platforms should establish explicit relationships among hydrogel architecture, MOF composition, microenvironmental responsiveness, degradation behavior, ion and cargo availability, resistance-associated mechanisms, and tissue regeneration. From a translational perspective, the most relevant systems will not necessarily be those exhibiting the highest antimicrobial activity, but those capable of providing a predictable therapeutic window, reproducible manufacturing, controlled degradation, validated resistance-modulating mechanisms, and acceptable long-term tissue compatibility [114].

Addressing these requirements will be essential for determining whether hydrogel–MOF composites can progress from multifunctional experimental materials to clinically relevant adaptive wound interfaces for the management of chronic infections and AMR.

## 10. Conclusions

Hydrogel–MOF composites have emerged as versatile wound interfaces, but their therapeutic performance is strongly influenced by MOF composition and the resulting structure–function relationships. Zn-based systems, particularly ZIF-8-containing platforms, have demonstrated significant potential through pH-responsive Zn^2+^ release, antibacterial activity, biofilm reduction, and support of angiogenesis and tissue regeneration. Cu-containing MOF systems provide additional advantages through bioactive metal-ion release and multimodal antibacterial mechanisms, including ROS-mediated and photothermal/photodynamic effects, whereas Zr-based frameworks such as UiO-66 and PCN-224 are particularly attractive due to their high structural stability and controlled cargo-delivery capabilities. Co-based and other catalytic MOF systems further expand therapeutic possibilities through oxidative mechanisms and synergistic antibacterial strategies. However, despite these advances, most reported hydrogel–MOF platforms remain primarily antibacterial and regenerative materials, with limited direct evidence demonstrating true modulation of antimicrobial resistance mechanisms.

Importantly, antimicrobial activity should not be equated with resistance modulation. Current evidence demonstrates that hydrogel–MOF systems can reduce bacterial viability, disrupt biofilm-associated structures, regulate oxidative and inflammatory processes, and facilitate localized therapeutic delivery. Nevertheless, direct evidence demonstrating inhibition of specific resistance mechanisms—including efflux-pump activity, HGT, QS-mediated adaptation, or resistance evolution—remains comparatively limited. Therefore, claims regarding resistance-modulating properties should be supported by mechanism-specific experimental validation rather than inferred solely from reductions in bacterial burden or biofilm biomass.

The integration of MOFs with hydrogel matrices provides a rational materials framework to address these challenges. MOF composition determines metal-ion availability, catalytic activity, cargo loading capacity, and stimulus responsiveness, whereas the hydrogel network regulates particle retention, transport, mechanical properties, and local therapeutic exposure. This hierarchical design enables the coordination of antimicrobial, antibiofilm, immunomodulatory, and regenerative functions according to the dynamic requirements of the wound microenvironment.

Future development of hydrogel–MOF systems will require a transition from empirically multifunctional materials toward mechanism-guided and quantitatively validated platforms. Greater emphasis should be placed on standardized evaluation of MOF degradation, ion/cargo release, microbial resistance pathways, host responses, long-term biosafety, and scalable manufacturing. Ultimately, the most clinically relevant hydrogel–MOF dressings may not be those exhibiting the highest nonspecific antimicrobial activity, but rather those capable of providing predictable therapeutic effects through validated mechanisms while selectively modulating the pathological processes responsible for chronic wound persistence.

## Figures and Tables

**Table 1:** Comparative overview of chronic wound microenvironmental features, AMR-associated mechanisms, and their clinical implications.

Category	Microenvironmental/Biological Feature	AMR-Associated Consequence	Clinical Implication	Ref.
Chronic wounds	Persistent inflammation, impaired healing, recurrent infection, altered physicochemical conditions	Sustained microbial persistence and repeated antimicrobial exposure may favor resistance and tolerance	Delayed healing, recurrent infection, increased morbidity and healthcare burden	[29,30,31]
Biofilm microenvironment	Polymicrobial communities, EPS protection, hypoxia, altered pH, oxidative stress, restricted antimicrobial diffusion	Reduced antimicrobial penetration, metabolic heterogeneity, phenotypic tolerance, and persistence	Treatment failure and recurrent infection	[4,29,32,33]
AMR-associated mechanisms	HGT, quorum sensing, efflux activity, persister cells, MDR phenotypes	Increased survival under antimicrobial pressure and potential dissemination of resistance determinants	Reduced antibiotic susceptibility and prolonged infection	[4,29,34]
Clinical challenges	Polymicrobial infection, therapeutic tolerance, recurrent infection, prolonged treatment	Persistence of resistant/tolerant populations despite conventional interventions	Delayed healing, prolonged hospitalization, increased treatment burden, and amputation risk	[29,35]

Abbreviations: AMR, antimicrobial resistance; EPS, extracellular polymeric substances; HGT, horizontal gene transfer; MDR, multidrug resistance.

**Table 2:** Comparative overview of hydrogel-based strategies for modulating pathological features of chronic wounds.

Pathological Feature	Biological Consequence	Hydrogel Strategy	Main Mechanism	Reported Effect	Key Limitation/Consideration	Refs.
pH imbalance	Sustained protease activation, impaired cellular activity, delayed healing	Chitosan- and alginate-based buffering hydrogels	Protonation/deprotonation and ionic exchange	Local pH stabilization and improved granulation tissue formation	Performance depends on buffering capacity, polymer chemistry, and wound pH dynamics	[33,45]
Excess ROS	Oxidative stress, cellular senescence, tissue damage	Antioxidant-loaded HA and gelatin hydrogels	ROS scavenging and redox regulation	Reduced oxidative injury and improved re-epithelialization	Antioxidant performance depends on ROS species, assay conditions, cargo, and release kinetics	[46,47]
Persistent inflammation	Elevated TNF-α/IL-1β and prolonged inflammatory signaling	Collagen- and HA-based immunomodulatory hydrogels	Cytokine regulation and macrophage modulation	Reduced inflammatory signaling and improved wound closure	Magnitude of immunomodulation depends on hydrogel composition and incorporated bioactive signals	[48,49]
Biofilm persistence and antimicrobial tolerance	Chronic infection, toxin release, reduced antimicrobial efficacy	Antibiofilm hydrogels incorporating chitosan, alginate, or bioactive agents	Electrostatic interactions and EPS destabilization	Reduced biofilm burden and bacterial viability in experimental models	Antibiofilm activity does not by itself demonstrate reversal of genetic AMR or restoration of antibiotic susceptibility	[46]
Excess exudate/proteases	ECM degradation and loss of regenerative signaling	Highly swelling alginate and cellulose dressings	Fluid absorption and protease sequestration	Improved moisture balance and reduced proteolytic damage	Excessive swelling may compromise dimensional and mechanical stability	[9,50]
ECM degradation	Loss of structural support and impaired cell adhesion	ECM-mimetic collagen and gelatin hydrogels	Integrin-mediated interactions and MMP modulation	Enhanced cell migration and matrix deposition	Performance depends on crosslinking, degradation rate, and scaffold mechanics	[9,39]
Hypoxia	Reduced angiogenesis and impaired tissue regeneration	Oxygen-permeable and oxygen-releasing hydrogels	Controlled oxygen diffusion and localized oxygen delivery	Improved oxygenation and angiogenic responses	Oxygen delivery must be balanced against dose, duration, material stability, and potential oxidative effects	[8,47]

Abbreviations: AMR, antimicrobial resistance; ECM, extracellular matrix; EPS, extracellular polymeric substances; HA, hyaluronic acid; IL-1β, interleukin-1 beta; MMP, matrix metalloproteinase; ROS, reactive oxygen species; TNF-α, tumor necrosis factor-alpha.

**Table 3:** Representative hydrogel–MOF systems with potential resistance-modulating effects relevant to efflux-mediated antimicrobial resistance.

Hydrogel–MOF System	Key Characteristics	Proposed Resistance-Modulating Mechanism	Reported Performance	Evidence Status	Refs.
NIF-doped ZIF-8 hybrid membrane	Sustained Zn^2+^ release, pH-responsive degradation, antibiofilm activity	Zn^2+^-mediated membrane perturbation and potential disruption of electrochemical gradients may increase antimicrobial susceptibility; direct EPI has not been established	Controlled and sustained Zn^2+^ release for 5 days, maintaining antibacterial activity against *S. aureus* and *E. coli*	Indirect; no direct EPI validation	[79]
GelMA@Sr-ZIF-8 hydrogel	Antibacterial, immunomodulatory, and pro-regenerative properties	Sustained metal-ion release may perturb bacterial homeostasis and contribute to membrane or metabolic stress; a direct effect on efflux-pump regulation remains unverified	Biphasic metal-ion release with antibacterial and antibiofilm effects	Indirect; no direct EPI validation	[77]
Hydrogel@ZIF-8@metronidazole	Controlled antibiotic release and localized infection management	High local antibiotic availability may increase intracellular drug exposure; saturation of efflux capacity remains a proposed mechanism rather than a demonstrated effect	Metronidazole adsorption capacity of 132.45 mg/g and accelerated release under acidic conditions (pH 5)	Proposed; direct EPI validation absent	[76]
Chitosan hydrogel/ZIF-8@PDA	Photothermal activity, antibacterial performance, and stimuli responsiveness	Photothermal and photocatalytic effects may increase membrane permeability and compromise bacterial transport processes; direct disruption of efflux complexes requires validation	~99% bacterial inhibition through combined ionic, photocatalytic, and photothermal effects	Indirect; antibacterial effect demonstrated	[80]
Hierarchical ZIF-8 bicomponent hydrogel	High drug-loading capacity and prolonged release profile	Sustained antibiotic exposure may increase intracellular drug availability, but prolonged exposure alone does not establish efflux-pump inhibition	92.07% cumulative BBH release at 24 h under acidic conditions (pH 5) against *S. aureus*	Proposed; direct EPI validation absent	[81]
Injectable alginate/gelatin hydrogel-Au@ZIF-8	Injectable, antibacterial, and wound-healing platform with controlled release	Light-induced ROS may damage bacterial membranes and proteins and thereby indirectly impair transport processes	Enhanced ROS generation under visible-light activation with antibacterial activity against *E. coli* and *S. aureus*	Indirect; no direct EPI validation	[82]

Abbreviations: MOF, metal–organic framework; NIF, nifedipine; ZIF-8, zeolitic imidazolate framework-8; GelMA, gelatin methacryloyl; PDA, polydopamine; ROS, reactive oxygen species; EPI, efflux pump inhibitor; BBH, berberine hydrochloride; Zn^2+^, zinc ion; Sr, strontium; Au, gold; *S. aureus*, *Staphylococcus aureus*; *E. coli*, *Escherichia coli*.

**Table 4:** Representative hydrogel–MOF systems with reported or proposed effects on EPS disruption and biofilm destabilization.

Hydrogel–MOF System	Biofilm Model	Proposed/Primary Mechanism of EPS Modulation	Potential Consequence for Biofilm Architecture	Resistance-Modulating Relevance	Reported Antibacterial Performance	Refs.
Chitosan–ZIF-8	*S. aureus*, *E. coli*	Electrostatic interactions with negatively charged EPS components and Zn^2+^-mediated ionic interactions	Reduced matrix cohesion and altered EPS organization	Increased accessibility of embedded bacteria to antimicrobial agents and immune effectors	Formulation containing 10% doxycycline and 4% ZIF-8 achieved inhibition zones of 22.5 mm against *S. aureus* and 14 mm against *E. coli*	[87]
Alginate/gelatin–ZIF-8	*S. aureus*, *E. coli*	Localized Zn^2+^ release and modulation of biofilm permeability	Increased matrix porosity and facilitated transport through biofilm layers	Enhanced penetration of therapeutic agents into mature biofilms	99% bacterial inhibition against *E. coli* and *S. aureus*; in vivo wound closure reached 89.40% by day 21	[88]
Bacterial cellulose–ZIF-8	Mixed-species biofilms	Hydration and microenvironmental modulation	Partial alteration of EPS hydration and polymer organization	Reduced biofilm stability and potentially increased treatment susceptibility	ZIF-8 crystals within the hydrogel measured 25–200 nm; inhibition zone of 35 mm against *P. aeruginosa*	[89]
PEG hydrogel–metal MOFs (Cu, Co, Zn)	*S. aureus*, *E. coli*	Controlled metal-ion release and catalytic oxidative stress	Modification and weakening of EPS-associated structures	Potential enhancement of biofilm dispersal and reduction of collective antimicrobial tolerance	Cu-MOF hydrogel achieved 99.9% antibacterial activity at the minimum bactericidal concentration; fibroblast cell death remained at 12% after 24 h	[90]

Abbreviations: MOF, metal–organic framework; ZIF-8, zeolitic imidazolate framework-8; Zn^2+^, zinc ion; Cu, copper; Co, cobalt; Zn, zinc; PEG, polyethylene glycol; EPS, extracellular polymeric substance; *S. aureus*, *Staphylococcus aureus*; *E. coli*, *Escherichia coli*; *P. aeruginosa*, *Pseudomonas aeruginosa*.

**Table 5:** Biofilm-associated factors influencing HGT and potential hydrogel–MOF intervention strategies.

Biofilm-Associated Factor	Contribution to HGT and AMR Dissemination	Potential Hydrogel–MOF Intervention	Potential Resistance-Modulating Effect	Refs.
High bacterial density and cellular proximity	Facilitates conjugation, plasmid transfer, and interspecies genetic exchange within biofilm communities	Hydrogel matrices may modify bacterial mobility and spatial organization, potentially reducing stable cell–cell interactions	Reduced opportunities for conjugative transfer and resistance-gene dissemination	[104]
eDNA accumulation	Provides structural support and can serve as a reservoir of ARGs available for natural transformation	eDNA sequestration or oxidative degradation through selected hydrogel–MOF systems may reduce the availability of extracellular genetic material	Potential reduction in the pool of transferable genetic material	[99]
EPS structural integrity	Stabilizes biofilm architecture and can protect extracellular genetic material and MGEs from environmental degradation	EPS modulation through osmotic, ionic, or oxidative mechanisms may increase matrix permeability and destabilize biofilm structure	Potential reduction of protected niches that facilitate persistence and genetic exchange	[96]
QS signaling	Coordinates biofilm maturation, collective adaptation, and intercellular communication associated with persistence and resistance	Selected MOF systems may interfere with bacterial signaling pathways or modify local physicochemical conditions that influence signaling	Potential attenuation of communication networks associated with biofilm development and resistance adaptation	[108]
Physicochemical stress gradients (hypoxia, acidic pH, oxidative stress)	Influence bacterial competence, adaptive responses, and stress-associated resistance evolution	Stimuli-responsive hydrogels may locally modulate pH, redox conditions, or molecular transport within the biofilm	Potential reduction of environmental conditions that favor persistence and resistance-associated adaptation	[104]

Abbreviations: AMR, antimicrobial resistance; ARGs, antibiotic resistance genes; EPS, extracellular polymeric substance; eDNA, extracellular DNA; HGT, horizontal gene transfer; MGEs, mobile genetic elements; MOF, metal–organic framework.

**Table 6:** Comparative assessment of representative hydrogel–MOF systems for resistance-modulating wound applications.

Hydrogel–MOF System	MOF/Active Component	Main Demonstrated Function	Resistance-Related Relevance	Experimental Evidence Reported	Main Advantage	Main Limitation	Evidence Level	Refs.
ZIF-8@porous hydrogel membrane	ZIF-8/Zn^2+^	Antibacterial, anti-inflammatory and pro-healing activity; controlled Zn^2+^ release	Zn^2+^-mediated antibacterial activity and reduced microbial adhesion may limit biofilm establishment	Infected full-thickness wound model; inhibited bacterial invasion and promoted wound closure, angiogenesis and collagen deposition	pH-responsive Zn^2+^ release combined with antifouling/omniphobic architecture	Does not directly demonstrate efflux-pump inhibition or reversal of antibiotic resistance	Indirect AMR relevance; in vivo evidence	[121]
CS/PEG/ZIF-8–cephalexin composite film	ZIF-8 + cephalexin	Controlled antibiotic delivery and antibacterial activity	Combines an antibiotic with a Zn-based MOF, potentially improving local antimicrobial exposure	Sustained cephalexin release after an initial burst; antibacterial activity against several bacterial species; high L929 fibroblast viability	Simple combination of antibiotic delivery and MOF-based antibacterial activity	No direct measurement of efflux activity, resistance-gene expression or susceptibility restoration	Indirect AMR relevance; in vitro evidence	[122]
CMC/TC@UiO-66 hydrogel film	UiO-66/Zr^4+^ + tetracycline	Controlled tetracycline release and antibacterial wound dressing	Localized antibiotic delivery may reduce systemic exposure, but AMR modulation was not directly demonstrated	Controlled release for 72 h; inhibition zones of 1.3 cm (*E. coli*) and 1.7 cm (*S. aureus*); good HFF-1 cytocompatibility	High framework stability and controlled antibiotic release	Primarily a drug-delivery platform; no direct evidence of resistance-mechanism modulation	Antibacterial; no direct AMR evidence	[123]
CS/PEG/GOx@Co-MOF hydrogel	Co-MOF + glucose oxidase	Synergistic antibacterial and wound-healing activity	Generates oxidative antibacterial stress and releases Co species; may interfere with bacterial homeostasis but direct AMR effects were not established	Enhanced activity against *S. aureus* and *E. coli* compared with chitosan hydrogel; wound-healing evaluation	Combines GOx-generated H_2_O_2_ with Co-MOF activity	Oxidative/metal-ion effects cannot be equated with efflux-pump inhibition	Indirect AMR relevance; in vivo evidence	[124]
NOCC/AHA–c-di-GMP@ZIF-8 injectable hydrogel	ZIF-8 loaded with c-di-GMP	Antibacterial immunotherapy and infected-wound repair through STING activation	Resistance is addressed indirectly by activating host innate immunity rather than targeting bacterial AMR machinery	Injectable hydrogel; c-di-GMP delivery through ZIF-8; infected-wound healing and STING-related immune activation	Couples MOF-mediated delivery with host immune activation	Does not directly target efflux pumps, HGT or resistance genes	Indirect AMR relevance; in vivo evidence	[125]
DMOG@PCN-224/ZIF-8 injectable hydrogel	PCN-224/ZIF-8 + DMOG	Chemo-photodynamic antibacterial therapy and angiogenic wound repair	Antibiotic-free antibacterial strategy potentially reduces dependence on conventional antibiotics	Rapid gelation (~30 s), controlled ROS therapy, antibacterial activity and long-term angiogenic effects reported	Antibiotic-free multimodal strategy with temporal therapeutic functions	AMR modulation remains a proposed benefit rather than a directly measured endpoint	Indirect AMR relevance; in vivo evidence	[69]
CA@ZIF-8/GGA injectable hydrogel	ZIF-8 + cinnamaldehyde	Sustained Zn^2+^/cinnamaldehyde delivery, angiogenesis and diabetic wound regeneration	Combines antibacterial/anti-inflammatory components, potentially reducing persistence-associated microenvironmental stress	Sustained release of CA and Zn^2+^; enhanced HUVEC proliferation, migration and tube formation; increased CD31/VEGF; accelerated diabetic wound closure	Integrates controlled MOF release with regenerative and anti-inflammatory activity	Study primarily addresses regeneration rather than direct AMR mechanisms	Indirect AMR relevance; in vivo evidence	[126]

Abbreviations: AMR, antimicrobial resistance; CA, cinnamaldehyde; c-di-GMP, cyclic di-guanosine monophosphate; CMC, carboxymethyl cellulose; CS, chitosan; DMOG, dimethyloxalylglycine; GOx, glucose oxidase; GGA, gallic-acid-grafted gelatin; HFF-1, human foreskin fibroblasts; MOF, metal–organic framework; NOCC, N,O-carboxymethyl chitosan; PCN-224, a zirconium-based porphyrinic MOF; PEG, polyethylene glycol; ROS, reactive oxygen species; STING, stimulator of interferon genes; ZIF-8, zeolitic imidazolate framework-8; Zn^2+^, zinc ion; Co-MOF, cobalt-based metal–organic framework; UiO-66, University of Oslo-66 zirconium-based MOF.

## Data Availability

Data sharing is not applicable to this article as no datasets were generated or analyzed during the current study.

## Supplementary Materials

The following supporting information can be downloaded at: https://www.mdpi.com/article/10.3390/gels12080744/s1, Figure S1. Schematic overview of bioactive hydrogel–MOF composites as resistance-modulating wound interfaces designed to disrupt biofilms, mitigate antimicrobial resistance, and promote regenerative wound healing. The illustration summarizes: (A) chronic wounds as reservoirs for the emergence and persistence of antimicrobial resistance; (B) bioactive hydrogels as microenvironment-modulating interfaces; (C) MOFs as molecular signal interceptors; (D) hydrogel–MOF systems for efflux pump inhibition (EPI); (E) extracellular polymeric substance (EPS) disruption and biofilm destabilization; (F) modulation of horizontal gene transfer (HGT) within wound biofilms; and (G) rational design of resistance-modulating bioactive hydrogel–MOF wound dressings integrating these complementary therapeutic mechanisms. Created in BioRender. Rizo, J. (2026) https://BioRender.com/71k1lza (accessed on 31 July 2026). Figure S2. Chronic wounds as reservoirs of antimicrobial resistance. Created in BioRender. Rizo, J. (2026). https://BioRender.com/s12f6va (accessed on 31 July 2026). Table S1. Representative metal-based MOFs for wound healing: biological functions, therapeutic potential, and molecular signal modulation. Table S2. Structural characteristics of representative MOFs discussed in Section 4. Figure S3. Conceptual illustration of the hierarchical construction and structural features of MOFs. The schematic depicts the progression from (A) fundamental building blocks (metal ions/clusters and organic linkers), to (B) MOF assembly through coordination-driven self-organization, (C) coordination chemistry and metal–ligand bonding environments, (D) development of pore architecture and framework topology, and (E) representative MOF families commonly investigated for biomedical applications. These structural characteristics govern porosity, stability, degradation behavior, cargo-loading capacity, and biological performance, making them key determinants of MOF functionality in wound-healing applications. Created using FigureLabs (https://chat.figurelabs.ai/verify/FL-PUB-20260803-FRC39U) (accessed on 31 July 2026). Figure S4. Design considerations for resistance-modulating biopolymer–MOF dressings, highlighting the influence of physicochemical properties, stimuli-responsive behavior, and biocompatibility on antimicrobial resistance control in chronic wounds. Created by the author using Canva (version 2.368.0). Table S3. Key design parameters governing the performance of resistance-modulating bioactive MOF wound dressings.
