# Supplementary material for: Bioactive Hydrogel–MOF Composites as Resistance-Modulating Wound Interfaces: Molecular Mechanisms and Rational Design for Chronic Wound Management
Source: Gels. 2026 Aug 20;12(8):744. doi: 10.3390/gels12080744 (PMC13512598; doi:10.3390/gels12080744)
Supplement: Supplementary file 1 [file gels-12-00744-s001.zip › gels-4405310-Supplementary.pdf]

Supplementary Information

Review

Bioactive Hydrogel–MOF Composites as Resistance-Modulating Wound Interfaces: Molecular Mechanisms and Rational Design for Chronic Wound Management

Nallely G. Hernández-Hernández [1], Irving A. González-Lara [1], Lesly Katleya Usme-Duque [1], Lía A. Martínez-Berlanga [1], Grecia D. Ortíz-Hernández [1,2], María I. León-Campos [1], Bertha Puente-Urbina [3], Miguel A. Medina-Morales [1], Elan I. Loredo-Alcalá [4], Leopoldo J. Ríos-González [1], Thelma K. Morales-Martínez [1], Roberto Arredondo-Valdés [1], Adolfo Romero-Galarza [1], Lucía F. Cano-Salazar [1], Rebeca Betancourt-Galindo [3], María O. González-Díaz [5,6], Nayeli Rodríguez-Fuentes [5,6], Javier Enríquez-Medrano [3], Florentino Soriano-Corral [3], Raul Rosales-Ibáñez [7], Amairany Rodríguez-Navarrete [7], Denis A. Cabrera-Munguía [1,*] and Jesús A. Claudio-Rizo [1,*]

[1] Facultad de Ciencias Químicas, Universidad Autónoma de Coahuila, Ing. J. Cárdenas Valdez S/N, República, Saltillo 25280, Coahuila, Mexico; katleya_usme@uadec.edu.mx (L.K.U.-D.); lia.berlanga@uadec.edu.mx (L.A.M.-B.); lucia.cano@uadec.edu.mx (L.F.C.-S.)
[2] Departamento de Horticultura, Universidad Autónoma Agraria Antonio Narro, Calz. Antonio Narro 1923, Buenavista, Saltillo 25315, Coahuila, Mexico
[3] Centro de Investigación en Química Aplicada (CIQA), Blvd. Enrique Reyna Hermosillo 140, San José de los Cerritos, Saltillo 25294, Coahuila, Mexico
[4] Center for Biodiversity Conservation and Ecology of Coahuila, Universidad Autónoma de Coahuila, Miguel Hidalgo 212, Zona Centro, Cuatro Ciénegas 27640, Coahuila, Mexico
[5] Centro de Investigación Científica de Yucatán (CICY), Calle 43 No. 130 × 32 y 34, Chuburná de Hidalgo, Mérida 97205, Yucatán, Mexico; nayeli.rodriguez@cicy.mx (N.R.-F.)
[6] Ministry of Science, Humanities, Technology and Innovation (SECIHTI), Av. Insurgentes Sur 1582, Crédito Constructor, Benito Juárez, Mexico City 03940, Mexico
[7] Tissue Engineering Laboratory and Translational Dentistry Clinic, Facultad de Estudios Superiores Iztacala, National Autonomous University of Mexico (UNAM), Tenayuca-Chalmita S/N, Cuautepec Barrio Bajo, Gustavo A. Madero, Mexico City 07239, Mexico; rosales_ibanez@unam.mx (R.R.-I.)
* Correspondence: dcabrera@uadec.edu.mx (D.A.C.-M.); jclaudio@uadec.edu.mx (J.A.C.-R.)

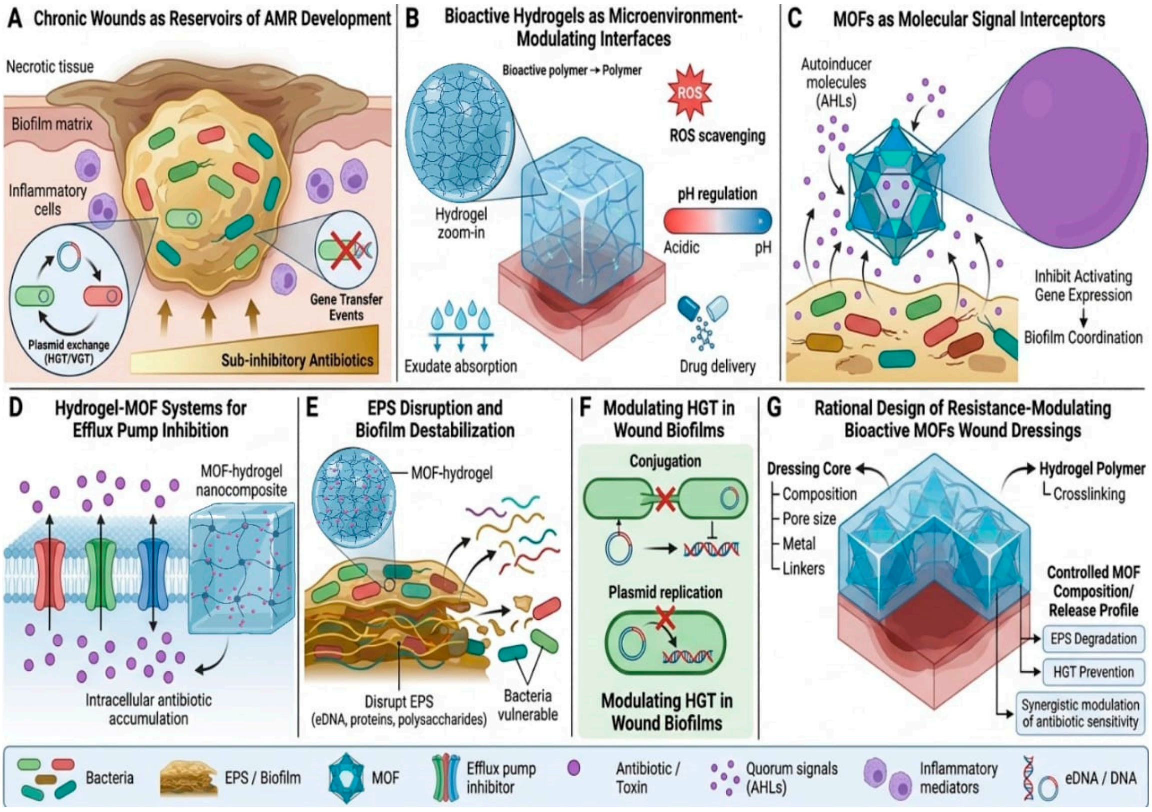

Figure S1. Schematic overview of bioactive hydrogel–MOF composites as resistance-modulating wound interfaces designed to disrupt biofilms, mitigate antimicrobial resistance, and promote regenerative wound healing. The illustration summarizes: (A) chronic wounds as reservoirs for the emergence and persistence of antimicrobial resistance; (B) bioactive hydrogels as microenvironment-modulating interfaces; (C) MOFs as molecular signal interceptors; (D) hydrogel–MOF systems for efflux pump inhibition (EPI); (E) extracellular polymeric substance (EPS) disruption and biofilm destabilization; (F) modulation of horizontal gene transfer (HGT) within wound biofilms; and (G) rational design of resistance-modulating bioactive hydrogel–MOF wound dressings integrating these complementary therapeutic mechanisms. Created in BioRender. Rizo, J. (2026) https://BioRender.com/71k1lza (accessed on 31 July 2026).

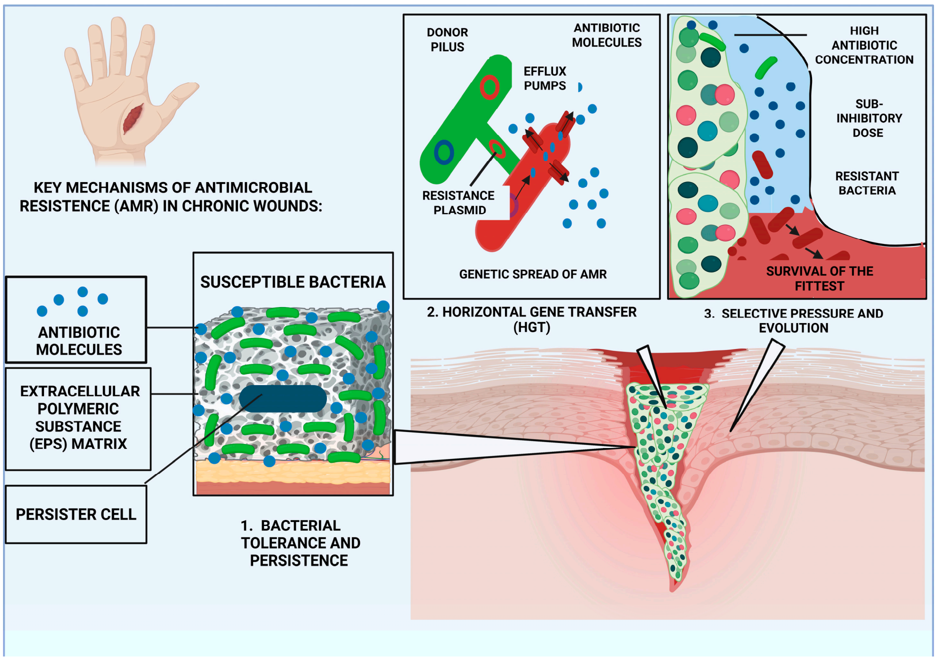

Figure S2. Chronic wounds as reservoirs of antimicrobial resistance. Created in BioRender. Rizo, J. (2026). https://BioRender.com/s12f6va (accessed on 31 July 2026).

Table S1. Representative metal-based MOFs for wound healing: biological functions, therapeutic potential, and molecular signal modulation.

Metal ion / representative MOF	Ligand & physicochemical behavior	Reported biological mechanisms/functions	Therapeutic relevance	Principal limitations	Refs.
Ag^+ / Ag-based MOFs	Azolate- or carboxylate-based frameworks; degradation and Ag^+ release depend strongly on ligand coordination and composite environment	Ag^+ disrupts thiol-containing proteins, membrane function, nucleic-acid processes, and microbial redox homeostasis; can increase ROS and interfere with biofilm-associated processes	Broad-spectrum antimicrobial activity; sustained/localized Ag^+ delivery; potential synergy with photothermal or other antimicrobial modalities	Therapeutic window is limited by host-cell toxicity, oxidative stress, and potential genotoxic effects at excessive Ag^+ exposure	[15]
Cu^{2+} / HKUST-1	Cu-BTC framework; relatively susceptible to degradation in aqueous/protein-rich environments; stability can be improved through surface	Cu^{2+} contributes to angiogenic signaling and ECM remodeling; Cu-based catalytic activity can promote ROS-mediated antimicrobial effects; HKUST-1 can additionally serve as	Combined antimicrobial, angiogenic, and regenerative activity; controlled Cu^{2+}/NO delivery can enhance diabetic wound repair	Rapid degradation and uncontrolled Cu^{2+} release may compromise cytocompatibility; therapeutic effects are strongly dose-	[55]

	modification or incorporation into matrices	a NO-delivery platform		and formulation-dependent	
Co^{2+} / ZIF-67	2-Methylimidazole-based framework; pH-dependent degradation and Co^{2+} release	Co^{2+} can induce a hypoxia-like response and increase HIF-1α-associated angiogenic signaling; can act synergistically with pro-angiogenic cargo	Potential enhancement of endothelial proliferation, migration, tube formation, and vascularization in poorly perfused wounds	Narrow therapeutic window; excessive Co^{2+} exposure may induce oxidative stress and cytotoxicity; long-term safety remains insufficiently established	[54]
Zn^{2+} / ZIF-8	2-Methylimidazole-based framework; acid-responsive degradation enables enhanced Zn^{2+} and cargo release under acidic conditions	Zn^{2+} contributes to cellular proliferation, enzymatic activity, antimicrobial responses, and tissue remodeling; pH-responsive degradation can couple pathological acidity to therapeutic release	Dual antimicrobial and regenerative potential; useful for responsive delivery of Zn^{2+} and therapeutic cargos	Excessive Zn^{2+} may impair cell migration and viability; direct causal links to specific signaling pathways require further validation	[53-75,76]
Fe^{2+}/Fe^{3+} / Fe-based MOFs	Fe-containing frameworks with tunable redox chemistry and degradation behavior	Fe redox cycling can regulate ROS generation/scavenging; Fe-based catalytic activity may contribute to antimicrobial effects and modulation of oxidative pathways	Potential control of oxidative stress and inflammatory microenvironment; catalytic activity can provide additional antimicrobial functionality	Redox activity can become detrimental at high Fe availability; Fenton chemistry and ferroptotic effects require careful dose control	[57]
Mg^{2+} / Mg-based MOFs	Mg-containing frameworks with variable aqueous stability and relatively favorable biological compatibility	Mg^{2+} participates in cellular metabolism, signaling, endothelial function, and tissue homeostasis; degradation may influence local ionic/pH conditions	Potential support of re-epithelialization, cellular migration, and regenerative processes	Limited direct antimicrobial activity; rapid degradation may reduce structural persistence and therapeutic control	[58]
Zr^{4+} / UiO- or porphyrinic Zr-MOFs	Highly stable Zr–O clusters; stability generally superior to	Primarily provide structural stability, high cargo retention, and, in porphyrinic systems,	Controlled delivery and photodynamic antimicrobial therapy; suitable	Zr centers are comparatively biologically inert; therapeutic	[56,106,123]

	many first-row transition-metal MOFs	photodynamic ROS generation under irradiation	for integrating multiple functionalities without relying primarily on metal-ion bioactivity	activity depends strongly on ligand/cargo functionality and external stimulation; synthesis cost may be high	
Ca^{2+} / Ca-based MOFs	Ca-containing frameworks generally exhibit comparatively rapid degradation in aqueous environments	Ca^{2+} can participate in calcium-dependent cellular signaling and ECM remodeling	Potential contribution to regenerative and tissue-remodeling processes	Rapid ion release may limit temporal control; evidence for direct modulation of chronic skin-wound signaling remains limited compared with other metal-based MOFs	[54,57]

Abbreviations: MOF, metal–organic framework; HKUST-1, Hong Kong University of Science and Technology-1; ZIF-67, zeolitic imidazolate framework-67; ZIF-8, zeolitic imidazolate framework-8; MIL-101, Materials of Institut Lavoisier-101; UiO, University of Oslo; TCPP, tetrakis(4-carboxyphenyl)porphyrin; BDC, 1,4-benzenedicarboxylic acid; BTC, 1,3,5-benzenetricarboxylic acid; VEGF, vascular endothelial growth factor; PDGF, platelet-derived growth factor; bFGF, basic fibroblast growth factor; NO, nitric oxide; HIF-1α, hypoxia-inducible factor-1 alpha; EPO, erythropoietin; NF-κB, nuclear factor kappa B; TNF-α, tumor necrosis factor-alpha; ALP, alkaline phosphatase; ECM, extracellular matrix; ROS, reactive oxygen species; POD, peroxidase; CAT, catalase; MDA, malondialdehyde; HIF-1α, hypoxia-inducible factor-1 alpha; CaM, calmodulin; CaMKII, calcium/calmodulin-dependent protein kinase II; MDR, multidrug-resistant.

Table S2. Structural characteristics of representative MOFs discussed in Section 4.

MOF	Metal node	Organic linker	SBU / coordination	Framework topology / pore characteristics	Representative wound applications	Refs.
ZIF-8	Zn^{2+}	2-Methylimidazolate	Tetrahedral ZnN_4	Sodalite (SOD), microporous (~11.6 Å cages)	Antibacterial, pH-responsive drug delivery, wound healing	[53]
ZIF-67	Co^{2+}	2-Methylimidazolate	Tetrahedral CoN_4	SOD topology, ZIF-8 analogue	ROS generation, antibacterial activity	[54]

HKUST-1	Cu^{2+}	Benzene-1,3,5-tricarboxylate (BTC)	Cu_2 paddlewheel SBU	3D porous framework with large cages	Cu-ion release, angiogenesis, antibacterial activity	[55]
UiO-66	$Zr_6O_4(OH)_4$ cluster	Benzene-1,4-dicarboxylate (BDC)	Zr_6 oxocluster	Highly stable fcu topology	Controlled drug delivery, tissue regeneration	[56]
MIL-100(Fe)	Fe^{3+}	BTC	Fe_3O cluster	Mesoporous cages (25–29 Å)	ROS generation, catalytic antibacterial activity	[57]
MOF-74 (CPO-27)	Mg^{2+} / Zn^{2+} / Co^{2+}	2,5-Dihydroxyterephthalate	Infinite metal–oxygen chains	One-dimensional channels (~11 Å)	Sustained metal-ion release	[58]

Abbreviations: MOF, metal–organic framework; SBU, secondary building unit; ZIF, zeolitic imidazolate framework; SOD, sodalite topology; BTC, benzene-1,3,5-tricarboxylate; BDC, benzene-1,4-dicarboxylate; DOBDC, 2,5-dihydroxyterephthalate; UiO, University of Oslo; HKUST, Hong Kong University of Science and Technology; CPO, coordination polymer of Oslo; ROS, reactive oxygen species.

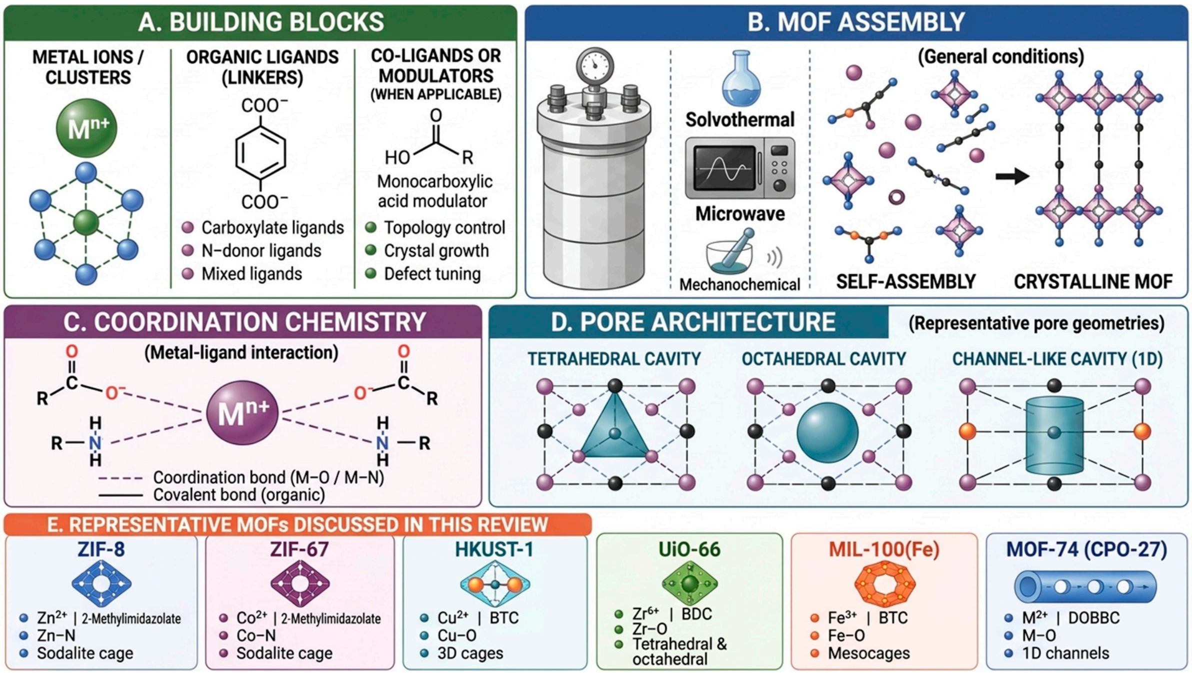

Figure S3. Conceptual illustration of the hierarchical construction and structural features of MOFs. The schematic depicts the progression from (A) fundamental building blocks (metal ions/clusters and organic linkers), to (B) MOF assembly through coordination-driven self-organization, (C) coordination chemistry and metal–ligand bonding environments, (D) development of pore architecture and framework topology, and (E) representative

MOF families commonly investigated for biomedical applications. These structural characteristics govern porosity, stability, degradation behavior, cargo-loading capacity, and biological performance, making them key determinants of MOF functionality in wound-healing applications. Created using FigureLabs (https://chat.figurelabs.ai/verify/FL-PUB-20260803-FRC39U) (accessed on 31 July 2026).

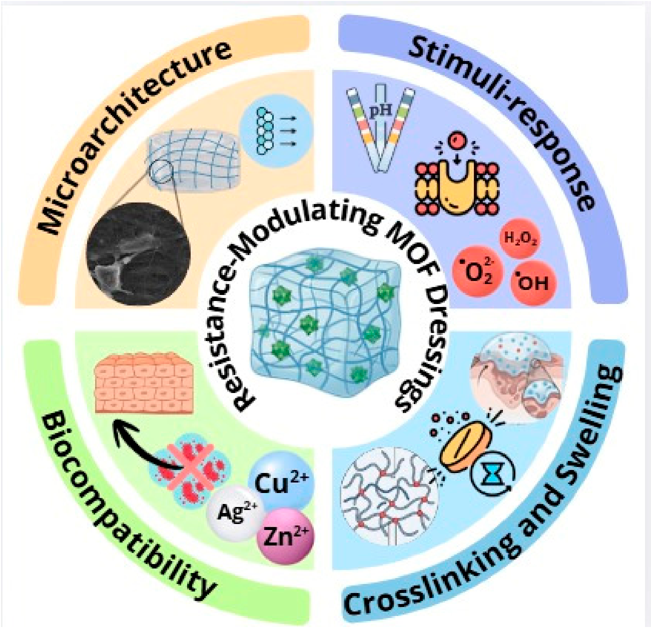

Figure S4. Design considerations for resistance-modulating biopolymer–MOF dressings, highlighting the influence of physicochemical properties, stimuli-responsive behavior, and biocompatibility on antimicrobial resistance control in chronic wounds. Created by the author using Canva (version 2.368.0).

Table S3. Key design parameters governing the performance of resistance-modulating bioactive MOF wound dressings.

Design parameter	Functional role in the dressing	Relevance for AMR modulation	Recommended performance metrics	Refs.
Crosslinking density	Regulates swelling, mesh size, degradation, mechanical stability, and therapeutic release	Controls the spatial and temporal availability of antimicrobial and resistance-modulating agents while maintaining dressing integrity	Swelling ratio; gel fraction; mesh size; degradation rate; release kinetics; mechanical retention during incubation	[39,42,63,112,113,114]
Stimuli-responsive behavior	Enables environmentally triggered changes in degradation, permeability, or	Enables localized treatment and may reduce unnecessary	Stimulus-dependent release ratio; response time; reversibility; release selectivity; stability	[21,39,41,52,75,78,114]

	cargo release in response to pH, ROS, enzymes, temperature, or other wound-associated stimuli	antimicrobial exposure by preferentially activating therapeutic functions under pathological conditions	under physiological conditions	
Porosity and network architecture	Governs fluid transport, oxygen diffusion, molecular transport, cellular infiltration, and MOF distribution within the hydrogel	Influences penetration of antimicrobial agents into biofilms and regulates the transport of ions and therapeutic cargoes within the wound interface	Pore-size distribution; pore interconnectivity; permeability; oxygen transport; swelling; cargo diffusion; biofilm penetration	[39,63,112,113,114]
MOF composition and metal-ion selection	Determines framework stability, degradation behavior, ion release, catalytic activity, and cargo-loading capacity	Defines the potential mechanisms for antimicrobial activity and resistance modulation, including biofilm disruption, redox regulation, efflux-pump interference, and modulation of microbial signaling	Metal-ion release kinetics; framework stability; cargo-loading efficiency; antimicrobial activity; biofilm disruption; mechanism-specific resistance assays; cytotoxicity	[53-55,60,75,106,114]
Mechanical properties	Provides structural integrity, flexibility, adhesion, and resistance to deformation during wound application	Maintains intimate contact with the wound and preserves localized delivery during mechanical stress and wound movement	Storage/loss modulus; tensile or compressive strength; toughness; adhesion; fatigue resistance; mechanical retention during degradation	[39,42,63,112,113,114]
Biocompatibility and biodegradability	Supports cellular viability and tissue remodeling while enabling controlled degradation and clearance	Defines the therapeutic window within which antimicrobial and resistance-modulating	Cell viability; inflammatory response; hemocompatibility where relevant; ion-release toxicity; degradation	[42,112,114,120,126,127]

		effects can be achieved without compromising host tissue	kinetics; degradation products; tissue compatibility	

Abbreviations: AMR, antimicrobial resistance; MOF, metal–organic framework; ROS, reactive oxygen species.
